# Global Variability in Reported Mortality for Critical Illness during the 2009-10 Influenza A(H1N1) Pandemic: A Systematic Review and Meta-Regression to Guide Reporting of Outcomes during Disease Outbreaks

**DOI:** 10.1371/journal.pone.0155044

**Published:** 2016-05-12

**Authors:** Abhijit Duggal, Ruxandra Pinto, Gordon Rubenfeld, Robert A. Fowler

**Affiliations:** 1 Institute of Health Policy, Management and Evaluation, University of Toronto, Toronto, Ontario, Canada; 2 Department of Critical Care, Respiratory Institute, Cleveland Clinic, Cleveland, Ohio, United States of America; 3 Trauma, Emergency and Critical Care Program, Sunnybrook Health Sciences Centre, Toronto, Ontario, Canada; 4 Interdepartmental Division of Critical Care and Department of Medicine, University of Toronto, Toronto, Ontario, Canada; University of Hong Kong, HONG KONG

## Abstract

**Purpose:**

To determine how patient, healthcare system and study-specific factors influence reported mortality associated with critical illness during the 2009–2010 Influenza A (H1N1) pandemic.

**Methods:**

Systematic review with meta-regression of studies reporting on mortality associated with critical illness during the 2009–2010 Influenza A (H1N1) pandemic.

**Data Sources:**

Medline, Embase, LiLACs and African Index Medicus to June 2009-March 2016.

**Results:**

226 studies from 50 countries met our inclusion criteria. Mortality associated with H1N1-related critical illness was 31% (95% CI 28–34). Reported mortality was highest in South Asia (61% [95% CI 50–71]) and Sub-Saharan Africa (53% [95% CI 29–75]), in comparison to Western Europe (25% [95% CI 22–30]), North America (25% [95% CI 22–27]) and Australia (15% [95% CI 13–18]) (P<0.0001). High income economies had significantly lower reported mortality compared to upper middle income economies and lower middle income economies respectively (P<0.0001). Mortality for the first wave was non-significantly higher than wave two (P = 0.66). There was substantial variability in reported mortality among the specific subgroups of patients: unselected critically ill adults (27% [95% CI 24–30]), acute respiratory distress syndrome (37% [95% CI 32–44]), acute kidney injury (44% [95% CI 26–64]), and critically ill pregnant patients (10% [95% CI 5–19]).

**Conclusion:**

Reported mortality for outbreaks and pandemics may vary substantially depending upon selected patient characteristics, the number of patients described, and the region and economic status of the outbreak location. Outcomes from a relatively small number of patients from specific regions may lead to biased estimates of outcomes on a global scale.

## Introduction

Mortality and morbidity associated with viral outbreaks is a major public health concern. With globalization, and increasing ease of travel these outbreaks have a higher likelihood to spread quickly and become significant global health threats. [1–4] The 2009–2010 Influenza A (H1N1) outbreak was declared the first pandemic of this century after early reports of high morbidity and mortality and subsequent imported cases and sustained transmission in many countries. [5–7]

Early outbreak reporting informs expectations in morbidity, mortality and guides health systems’ response, to specific outbreaks. [5, 6] This reporting is instrumental for public health risk assessments. [8] However, detection bias towards identification and inclusion of the sickest patients and selection bias (of specific sub-groups of patients) are substantial threats to the internal validity and generalizability for early outbreak studies. [9–11] As well, there may be unique geographic or socioeconomic characteristics of the first affected regions that influence patient outcomes. [5] Oftentimes, there is incomplete reporting of the entire outbreak period and population, leading to inaccurate estimates for future events.

The H1N1 pandemic was unique as it was the first instance of a pandemic that was widely reported over different geographical and socioeconomic strata at a global level during 2009–2010. [2, 12] However, influenza A (H1N1) comprised the majority of global seasonal influenza-related illness again in 2013–2014. [13] With this in mind, we performed a systematic review of all studies describing critically ill patients in the 2009–2010 Influenza A (H1N1) outbreak in order to better understand how patient, temporal, healthcare system and study-specific factors influence reporting of clinical outcomes and to attempt to provide unbiased and valid estimates of mortality. We deliberately did not focus upon all patients with H1N1 as there is no globally valid denominator to describe these patients over different geographic regions. Thus using a standardized definition of critical illness we included such patients across different geographic regions and time periods. Moreover, a focus on critically ill patients is clinically relevant as they are most likely to have poor outcomes and therefore represent an important group to target for early prediction and care.

## Methods

### Search Strategy

We searched Medline (January week 1, 2009 to March week 2, 2016), Embase Classic + Embase (2009 week 1 to 2016week 10), LILACS and African Index Medicus for studies that evaluated mortality associated with critical illness in confirmed, probable or suspected cases of 2009–2010 Influenza A (H1N1) infection (for detailed search strategy see S1 File). We reviewed the references of all retrieved studies and reviews to identify any additional studies. We considered full text articles published in any language. We did not consider abstracts or other material presented at medical conferences or unpublished data. The research and ethics committee of our institution waived the need for patient-level consent for this study as only aggregate and previously published data was collected.

### Study Selection

**Fig 1:**
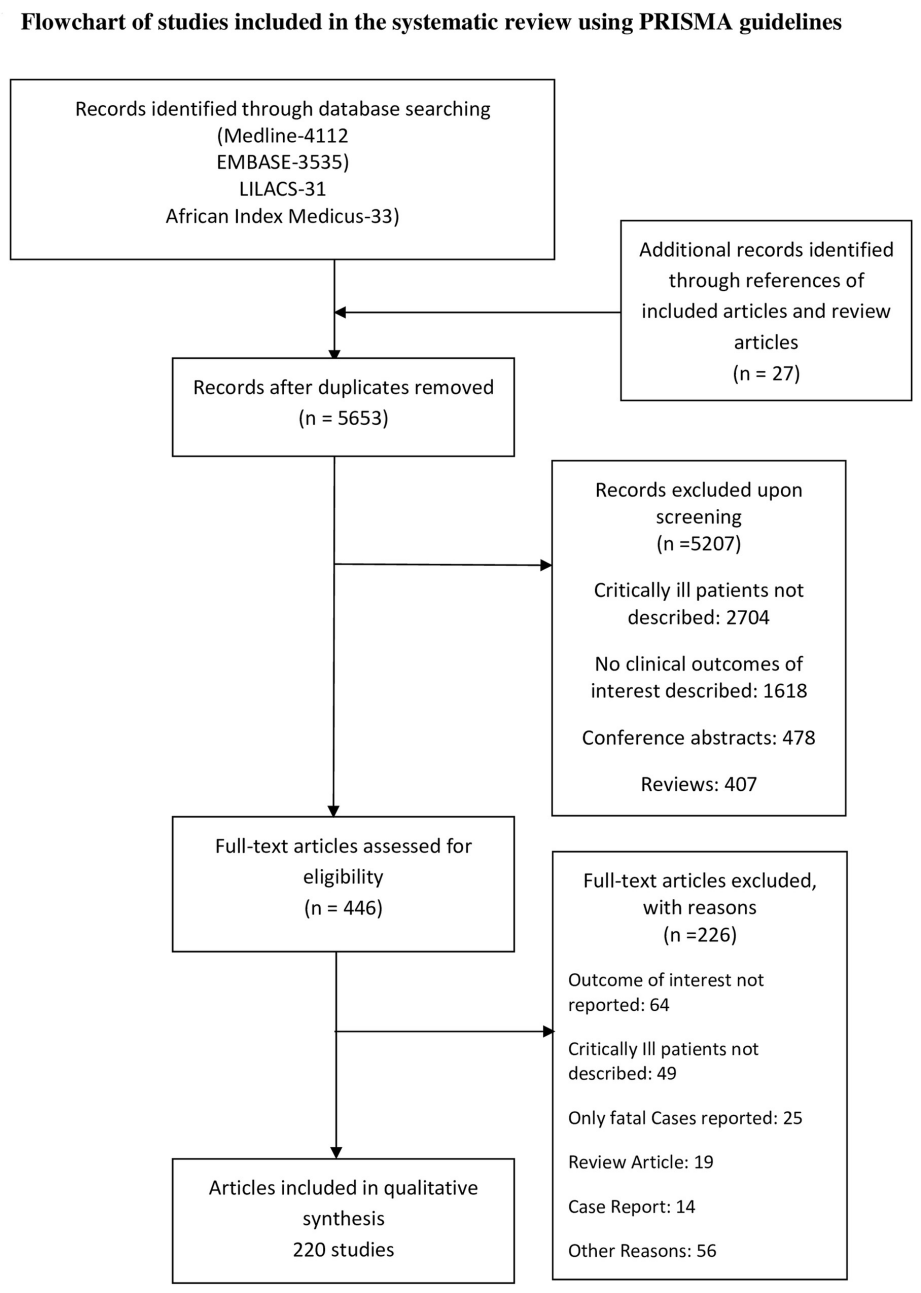
Preferred reporting items for systematic reviews and meta-analyses (PRISMA) flow diagram. Study identification and selection process.

Inclusion Criteria: (1) described confirmed, probable or suspected cases of 2009–2010 influenza A (H1N1) infection; and (2) described patient(s) who were critically ill (admission to an adult or pediatric intensive care unit (ICU) or area of the hospital where critically ill patients routinely receive treatment; or, patients receiving invasive or non-invasive mechanical ventilation; or, patients receiving continuous intravenous vasoactive medications; or, other criteria with justification presented in the individual study to designate patients as critically ill). Exclusion Criteria: (1) case series describing fewer than 5 patients; (2) studies that did not report mortality in critically ill patients, or only described characteristics of patients who died. Fig 1 outlines the study flow diagram based on PRISMA guidelines. [14] We anticipated that many early and potentially smaller studies would describe patients subsequently included in multicenter or national studies. To prevent non-independent reporting, we included studies only representing non-overlapping patient populations for the description of outcomes over different geographical or economic regions and specific ICU populations; however we included studies with potentially duplicated patients for description of outcomes over time, and for single versus multiple centers comparisons (Inclusion criteria for subgroups described in S1 File; Fig A in S1 File).

### Objectives

The primary objective was to determine mortality of critically ill patients with Influenza A (H1N1) during the 2009–2010 pandemic. We preferentially used hospital, then 1 month, then in-ICU mortality, whichever represented the longest period of follow-up in order to obtain the least time-biased estimate. Our secondary objective was to determine how study, healthcare system and patient-associated factors influence reporting of mortality.

### Data Extraction

Study characteristics and key results were abstracted by one author (AD) using a standardized study report form. The primary outcome was abstracted in duplicate from all studies by a second author (RF) to ensure a high degree of inter-rater reliability. We collected geographic (country, hemisphere, region and continent) variables and economic (World Bank designation) designation for each country [15]; whether the study included unselected (consecutive) or selective (non-consecutive) critically ill patients, or specific patient populations (e.g. adults and separately pediatric patients, only mechanically ventilated patients, only patients receiving rescue oxygenation therapy, only those with specific organ injury such as acute respiratory distress syndrome [ARDS] or acute renal injury); the duration of the study (based on the months and year of inclusion of the first and last patients of the study) and also whether the study period reported on the region-specific first wave, second wave, third wave or more than one wave of the pandemic.

We collected patient demographics reported in aggregate, severity of illness using the Acute Physiology and Chronic Health Evaluation (APACHE) II/III/IV, Pediatric Risk of Mortality (PRISM) II/III, sequential organ failure assessment (SOFA) or Simplified Acute Physiology Score (SAPS)II/III; age (overall, and among adults and children <18 years); sex; co-morbidities including obesity, diabetes, congestive heart failure, cerebrovascular disease, neoplastic disorders, chronic liver, or renal diseases; and the presence of immunosuppression; and, co-presenting conditions such as pregnancy or post-partum status (detailed definitions of all variables provided in S1 File).

### Quality Assessment

We used the Newcastle-Ottawa scale to assess study quality. [16, 17] The scale allocates up to 9 points to evaluate the risk of bias (higher scores indicate a smaller likelihood of bias) in cohort or case-control studies in 3 domains: selection of study groups (4 points), comparability of groups (2 points), and ascertainment of either exposure or outcome (3 points). As we were not comparing two distinct groups of patients we evaluated the risk for under- or over-reporting of mortality based on the three domains of the scale. We used a modified Newcastle-Ottawa Scale to assess the appropriateness of selection, and follow up of these patients and defined the risk as being high for studies with a score of 6 or lower.

### Statistical Analysis

We combined data from studies to estimate the pooled risk for death. We explored clinical heterogeneity by establishing subgroups of studies according to distinct patient populations and determined statistical heterogeneity among studies by using the Q statistic and I^2^ index. [18] We used a random effects model to obtain summary outcome point estimates and 95% confidence intervals. [19]

We conducted random effects meta-regression analyses for mortality for different variables extracted from the studies, including specific pandemic time periods (first wave, second wave, prolonged enrollment), geographical region (country, region, continent, World Bank economic development status), study population characteristics (unselected vs. selected patient populations, mechanically ventilated vs. not ventilated patients) and patient population factors (adults, children), pregnancy or post-partum state, specific illnesses (ARDS, acute kidney injury) and ICU specific interventions such as receipt of rescue oxygenation therapy (extracorporeal membrane oxygenation (ECMO), high frequency oscillatory ventilation (HFOV)). We also performed a paired analysis for all the countries that reported mortality both during Wave I of the pandemic as well as enrolled for longer than nine months. [20, 21] [22] Risk of publication bias was assessed with a funnel plot, Trim and fill method was used to identify and correct for funnel plot asymmetry. Meta-analysis and meta-regression exploring the influence of patient, study and health system factors on reported mortality was conducted on the logit transformed proportions. Analyses were performed using R version 3.2.5 and SAS version 9.4 (SAS Institute, Cary, NC).

## Results

### Study Flow

Our search strategy yielded 5653 citations after de-duplication. We retrieved 446 articles for a detailed evaluation and included 220 articles for our qualitative assessment. We identified 226 studies from 220 articles (six of the articles compared two different time periods of the pandemic and were thus reported separately) from 50 countries that met our inclusion criteria (Table 1). 115 studies with non-overlapping patients were used in the different meta-regression subgroups.

**Table 1:** System and study based characteristics described in 226 studies compared to the studies selected for the meta-regression and hierarchical model respectively.

Study Characteristics	All Studies (n-226)
Period of Enrollment	
April 2009-August 2009	50 (22%)
September 2009-January 2010	31 (14%)
February 2010 until end of pandemic	3 (1%)
Studies enrolling through different waves of the pandemic	144 (63%)
Multicenter Studies	109 (49%)
Study size (number of patients)	
5–10	36 (16%)
11–25	79 (35%)
26–100	68 (30%)
101–250	22 (10%)
>250	21 (9%)
Studies with only adult patients	140 (63%)
Studies describing unselected critically ill patients	155(69%)
Studies describing specific subgroups	
Mechanical ventilation	63 (28%)
ARDS	59 (26%)
Acute kidney injury	10 (4%)
Pregnant critically ill	8 (4%)
ECMO	20 (9%)
**Study geographical region**	
**Americas**	
North America*	43 (19%)
Latin America and Caribbean	26 (11%)
**Europe**	
Western Europe	69 (31%)
Eastern Europe	11 (5%)
**Asia**	
Middle East	12 (5%)
South Asia	12 (5%)
East Asia and Pacific	32 (14%)
**Africa**	
North Africa	3 (1%)
Sub-Saharan Africa	3 (1%)
**Australia/New Zealand**	16 (7%)
Study country economic status of the country	
High income economy	161 (71%)
Upper middle income economy	50 (22%)
Lower middle income economy	13 (6%)

Values are numbers (percentages) unless stated otherwise. We describe the system based, temporal and geographical characteristics of countries included in our systematic review. We also describe similar variables for studies included in our meta-regression and our hierarchical model. This table shows that at each level the relative distribution of the variables remained constant throughout the reported studies.

*Mexico is analyzed separately from United States and Canada and is considered to be a part of Latin America and Caribbean according to World Bank geographical regions. ARDS: Acute Respiratory Distress Syndrome; ECMO: Extracorporeal Membrane Oxygenation

### Study Characteristics

The study characteristics were similar when combined for both the meta- regression and the hierarchical meta-regression (Table A in S1 File). There was no clinically important difference in the reporting of demographic, and intervention variables among populations when we evaluated all included studies compared to studies only included in the meta-regression (Table 2). Median age of patients was 40 (32–44) years, 49% were female, Among the 89 (39%) studies, which reported APACHE II scores, the median APACHE II was 18 (14–21). 132 (58%) studies reported on the incidence of ARDS, and the median incidence of ARDS was 95% among those studies.

**Table 2:** Description of patient characteristics, intensive care specific interventions and outcomes from included studies compared to the studies selected for the meta-regression respectively.

Characteristics	All studies (n = 226)		Studies for meta-regression (n = 115)	
	*N*	Median (IQR), Proportion	*N*	Median (IQR), Proportion
Age	*179*	40 (32–44)	*88*	42 (37–46)
Females	*177*	49%	*89*	49%
APACHE II	*89*	18 (14–21)	*44*	17 (14–19)
Pre-existing lung disease	*144*	26%	*74*	25%
Obesity	*102*	29%	*62*	27%
Pregnancy	*103*	9%	*59*	9%
**ICU Complications**				
ARDS	*132*	95%	*74*	98%
Acute renal failure	*49*	36%	*25*	39%
**ICU Treatments**				
Renal replacement therapy	*63*	17%	*35*	16%
Inotropes	*98*	50%	*47*	51%
Antivirals	*92*	100%	*53*	100%
Antibiotics	*49*	100%	*27*	100%
Corticosteroids	*70*	48%	*32*	52%
**Outcomes**				
Duration of mechanical ventilation	*74*	10 (8–14)	*38*	10 (7–14)
ICU length of stay	*99*	11 (8–18)	*49*	11 (8–20)
Mortality*	*226*	28%	*115*	32%

Categorical variables are described as numbers (percentages) and continuous variables are described as median (interquartile range) unless stated otherwise. N Denotes the number of studies that reported on each variable. The reporting of patient level variables remained similar at all levels of our analysis of the reported studies. APACHE II: Acute Physiology and Chronic Health Evaluation II; ARDS: Acute Respiratory Distress Syndrome; ICU: Intensive Care Unit.

*The mortality described is a mean of all studies reporting short term mortality associated with critical illness during the H1N1 pandemic

### Risk of Bias and quality of evidence assessment

We did not identify any randomized controlled trials; therefore, only observational studies (cohort, case-control) were included in our analysis. The media Newcastle-Ottawa Scale score was 7 (range 4–9). Most studies were considered to be of high quality (Table B in S1 File). The funnel plot displayed visual asymmetry (Fig B in S1 File) and the trim and fill analysis estimated 15 missing studies. (Fig C in S1 File) Based on the Egger’s classical test (p = 0.026) the publication bias was statistically significant.

### Meta-Regression of reported mortality during the 2009 Influenza A (H1N1) pandemic

After excluding duplicate studies and those reporting exclusively on pediatric patients we extracted data from 115 studies to report on the mortality associated with specific subgroups of patients.

### Reported mortality over time

**Fig 2:**
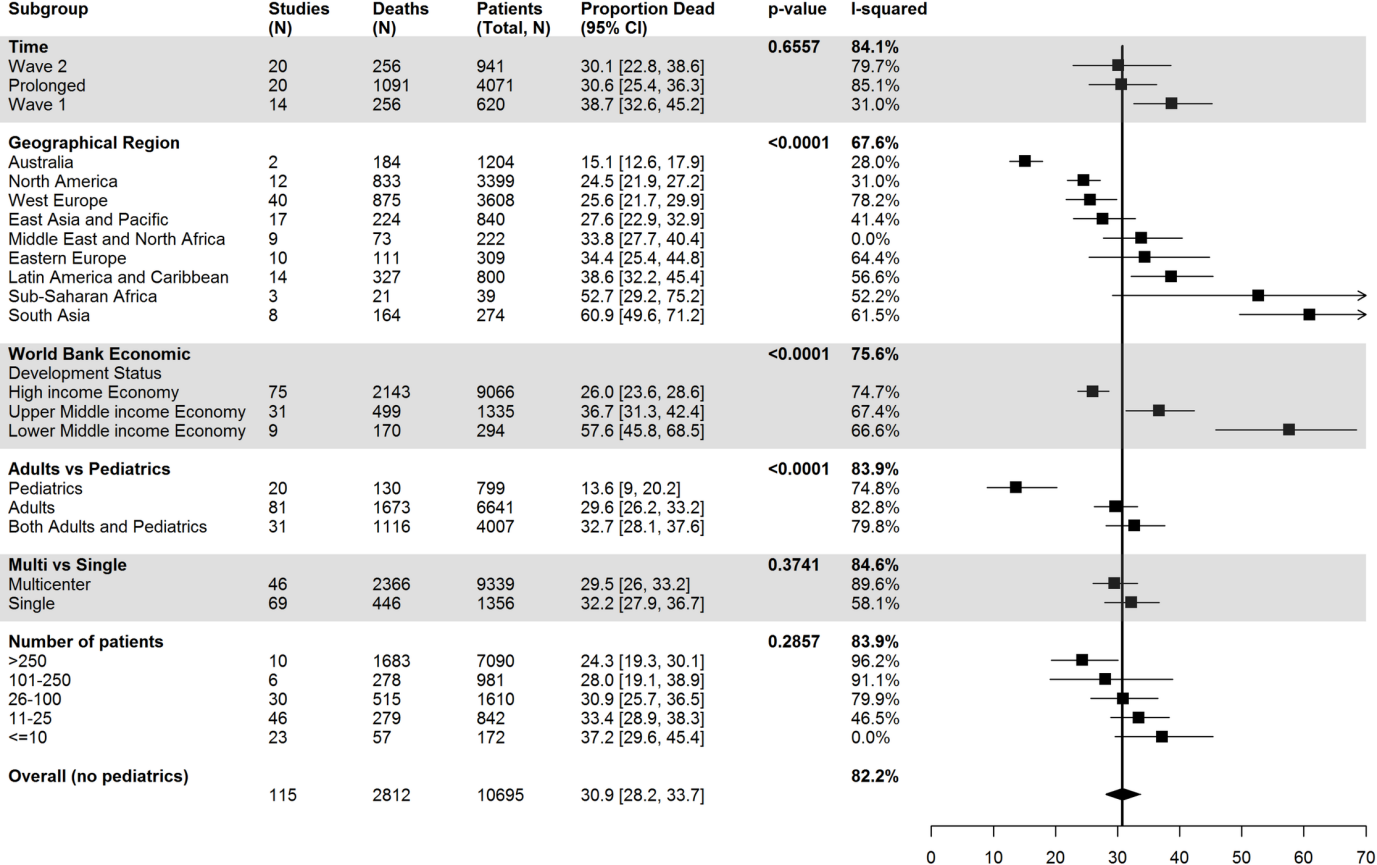
Reported mortality associated with 2009 Influenza A (H1N1) associated critical illness. We describe the mortality based on temporal (early, late and prolonged enrollment), study (study size, single center compared to multicenter and adults compared to pediatrics), and the geographic location and socioeconomic development from the included studies. The black squares represent the point estimate and 95% confidence intervals (CIs) around the mortality for each subgroup. The black diamond is the summary or overall combined estimate of mortality associated with the 2009 Influenza A (H1N1) pandemic.

14 studies reported during the first wave of the pandemic, and 20 studies reported on the second wave. Nineteen manuscripts described patients over a prolonged period of time (>9 months). Overall mortality was 31% among adult patients. Mortality for the first wave of the H1N1 pandemic was 39% (95% CI 33–45), 30% (95% CI 23–39) in wave 2, and 31% (95% CI 25–36) during prolonged enrollment (p = 0.66). (Fig 2) When analyses was restricted to those countries that reported on early as well as prolonged periods of time from within the same country, there was no difference in mortality. (Table 3)

**Table 3:** Meta-analysis comparing the reported mortality from “early enrollment” (the Wave 1 for each individual country) during the H1N1 pandemic with studies describing prolonged enrollment from the same countries. We evaluated the differences in the reported mortality among studies from individual countries by using both a fixed-effects and a random-effects model.

Country	Relative Risk (95% CI)
Australia/ New Zealand	1.09 (0.87–1.36)
Canada	1.27 (0.89–1.82)
China	1.3 (1.01–1.68)
France	2.66 (0.89–7.9)
Italy	1.003 (0.28–3.53)
Mexico	0.85 (0.42–1.74)
Spain	0.84 (0.45–1.55)
USA	0.77 (0.51–1.16)
Random Effects Model	1.11 (0.93–1.31
	p-value = 0.23
Quantification of Heterogeneity	Q = 8.92
Test of Heterogeneity	I^2^ = 21.6% (0%-64%)
d f = 7	p-value = 0.26

The table shows that at an individual country level, the relative risk of death was not statistically significantly different during the duration of the pandemic. The reporting from early case-series gave an approximate estimate of the overall mortality in any given country though the entirety of a pandemic. However, we also found that there were significant intra-country differences in the reported mortality among different countries, and these differences also tended to remain constant when they are studied through the entirety of the pandemic.

### System-based Variables

#### Geographical area of the study

**Fig 3:**
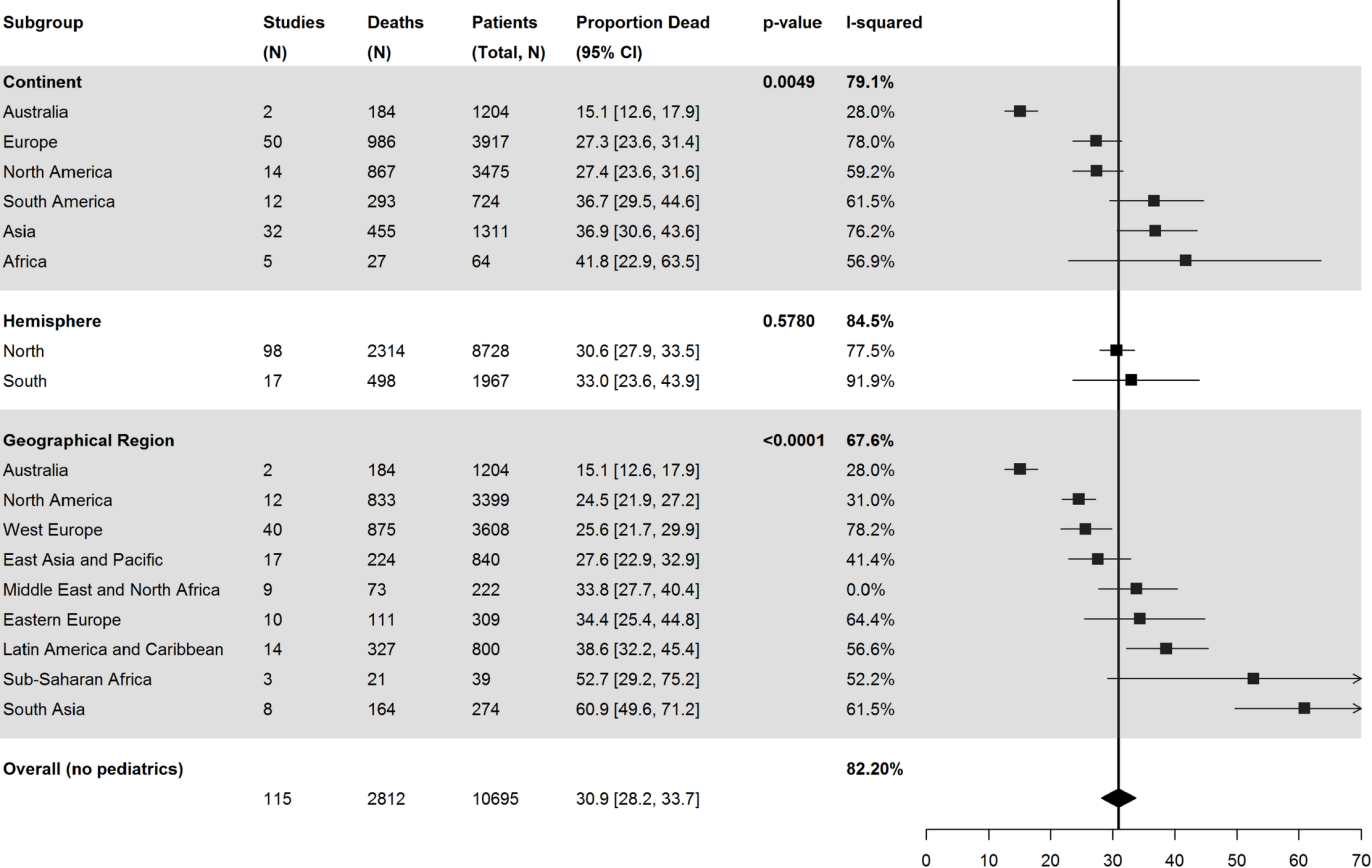
Differences in reported mortality based on different geographic variables for the included countries (hemisphere, continent and World Bank designated geographical region). The Black squares represent the point estimate and 95% confidence intervals (CIs) around the mortality for each subgroup. The black diamond is the summary or overall combined estimate of mortality associated with the 2009 Influenza A (H1N1) pandemic. The use of geographical regions is associated with the best discriminative power to report the differences in mortality in a global context.

There was no difference in the mortality based on studies from northern hemisphere (31% [95% CI 28–34]) compared to the southern hemisphere (33% [95% CI 24–44]); however, we found significant differences among different continents and geographical regions (Fig 3). Continental mortality reported from Australia was lowest (15% [95% CI 13–18]), from Africa the highest (42% [95% CI 23–64]), from Asia 37% (95% CI 31–44), South America (36% [95% CI 29–45]), North America 27% (95% CI 24–32), and Europe 27% (95% CI 23–31). When we compared the reported mortality based on the geographical region mortality was the highest in the South Asian (61% [95% CI 50–71]) and Sub-Saharan African (53% [95% CI 29–75]) countries. Mortality was comparable in North America (25% [95% CI 22–27]); West Europe (25% [95% CI 22–30]) and East Asia and Pacific 28% [95% CI 23–33). Reported mortality in Middle Eastern and North African countries (34% [95% CI 28–40]), Eastern European (35% [95% CI 26–47]), and Latin American Countries (39% [95% CI 32–45]) all showed a more pronounced effect when the geographical region rather than the hemisphere or the continent was considered.

#### Economic status of the country

High income economies had significantly lower reported mortality (26% [95% CI 24–29] compared to upper middle income economies (37 [95% CI 31–42]) and lower middle income economies (58% [95% CI 46–69]) respectively (P<0.0001). (Fig 3) There were clinically relevant differences in the duration of mechanical ventilation among studies from high income economies (11[8–16] days) compared to upper (9 [8–10] days) and lower (8 [6–10] days) middle income economies but not in ICU length of stay (Table B in S1 File).

### Patient-related Factors

#### Age

Mortality was significantly lower in the pediatric studies (14% [95% CI 9–20]) compared to the adult studies (30% [95% CI 26–33] (P<0.0001) in unselected critically ill patients.

#### Specific patient populations

**Fig 4:**
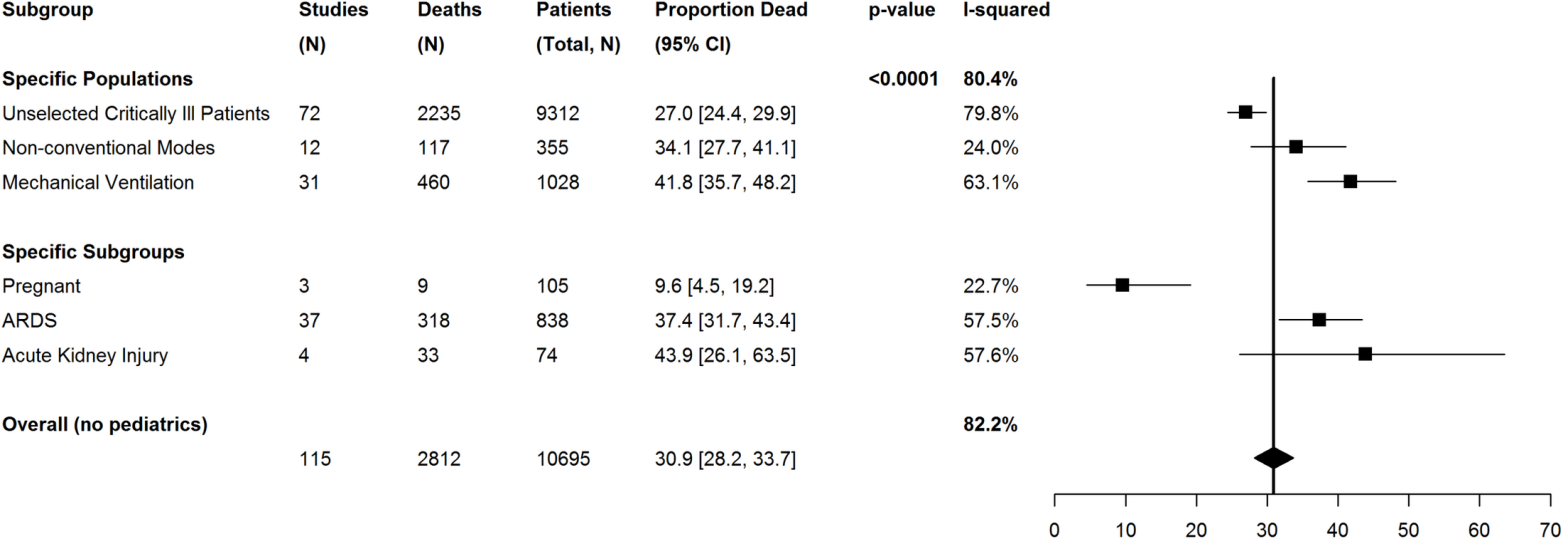
Differences in reported mortality based on subgroups of patients with different severity of illness (need for mechanical ventilation), critical illness associated organ failure (ARDS; AKI) or co-presenting conditions (pregnancy). The black squares represent the point estimate and 95% confidence intervals (CIs) around the mortality for each subgroup. The black diamond is the summary or overall combined estimate of mortality associated with the 2009 Influenza A (H1N1) pandemic

Mortality was substantially higher among patients undergoing mechanical ventilation (42% [95% CI 36–49]) in comparison to unselected critically ill patients, (27% [95% CI 24–30]) (Fi 4). Mortality in patients with ARDS was 37% (95% CI 32–44) and 44% [95% CI 26–64] among critically ill patients with acute kidney injury, and 10% (95% CI 5–19) among critically ill pregnant patients (Fig 4; Table C in S1 File).

## Discussion

In this systematic review and meta-regression of 226 studies investigating pandemic influenza A (H1N1)-related critical illness from 50 countries, we found that overall adult mortality was 31%. There was substantial variability in mortality according to the global region and the country’s economic development status–mortality in sub-Saharan Africa and South Asia was double that of Australia, North America and Western Europe. Studies of specific populations and those from early in the pandemic were smaller, clinically more heterogeneous and had a higher reported mortality. There were significant differences in reported mortality based on the severity of illness. The need for mechanical ventilation and critical illness associated acute kidney injury were most strongly associated with higher reported mortality at a global level.

These findings are important because they emphasize that patient specific factors such as organ failure or need for artificial life support, along with geographic area or economic development of a country from which these events are being reported can have a significant influence on the reported mortality during disease outbreaks. These findings highlight the limitations to generalizing early reported outcomes from a limited region and among a relatively small number of patients and have relevance for contemporary outbreaks such seasonal and avian influenza, Middle East Respiratory Syndrome Coronavirus and Ebola.

Based on our findings early reports during outbreaks and pandemics should ideally describe consecutively enrolled, objectively defined but minimally selected patients to best inform appropriate clinical and policy decisions. These reports should be separate from publications reporting on selected populations. Such reports are important to identify risk factors for differential outcomes, but it is very important that such populations should be clearly defined. Such differentiation ensures an accurate and homogenous assessment of disease severity at a global scale. This allows for early recognition in differences in outcomes associated with disease outbreaks over different time periods and geographical regions when considering its impact at a global scale. Ideally this would be accomplished using prospectively developed, flexible and tiered case report forms that are appropriate for a variety of resource settings.[23]

Reporting on differences in regional outcomes associated with critical illness in a global context is challenging. The lack of standardized definitions, and differences in severity of disease recognized as critical illness have been cited as potential barriers. [24–26] When we compared differences in reported mortality based on early reporting compared to prolonged periods of enrollment for countries with available data, there was no difference in the reported mortality over time among countries, but country-specific differences in reported mortality persisted over time (Table 3). [7, 27–29] Our study highlights that the use of geographic variables such as hemispheres or continents is likely less sensitive to differences among regions as differential resources and patient characteristics can exist within broadly defined geographical units. The use of either economic development or geographic regions as defined by World Bank was more sensitive in demonstrating the impact on reported mortality. The economic development of the country might be a surrogate marker for the availability of ICU specific therapies, recognizing that substantial intra-country differences may exist among private and public systems.

Strengths of this systematic review include a comprehensive search strategy, with duplicate screening and outcome data abstraction, providing the most complete review of pandemic H1N1 outcomes to date. We used validated strategies to minimize bias in the selection of studies and reporting of outcomes and *a priori* planned the combinations of studies to report on different time periods of the pandemic, specific sub-groups and clinically important interventions. We used meta-regression to examine the impact of moderator variables on study effect size. We used random-effects models to aggregate data and generate conservative confidence limits for point estimates. We used the Newcastle-Ottawa scale to ensure we adequately recognized and reported the risk of bias. Although we included studies from all regions of the world, the majority of our outcomes were informed by developed regions with capacity to carry out observational and intervention research, and also, the capacity to provide tertiary care for these patients. Because most prior estimates of global mortality have used extrapolated mortality rates from high-income countries, it is quite possible that global pandemic-associated mortality is higher than previously reported. [12, 30]

## Conclusions

In this systematic review of the published literature examining global patient characteristics and outcomes for H1N1-related critical illness during the 2009–2011 pandemic, we provide the most accurate and valid estimates of outcomes, and explore how these outcomes differ according to population, patient and study characteristics. Reported mortality for new outbreaks may vary substantially depending upon selected patient characteristics, the number of patients described, and the region and economic status of the outbreak location. These findings have relevance for new and ongoing outbreaks. Outbreaks should use case report forms that are prospectively developed, modifiable depending upon the illness syndrome, scalable to a variety of resource settings, encompass some measure of severity of illness to allow for risk adjustment across regions, and should be freely and globally available. [23] A standardized global approach to reporting on outbreaks and pandemics will provide us more accurate estimates of morbidity and mortality associated with new diseases and provide to the most valid information upon which to base current and future research, clinical care, and health systems responses.

## Supporting Information

S1 File Supplement for H1N1 global variability manuscript. **Fig A:** Flowchart for subgroups of studies analyzed in the meta-regression. **Fig B:** Funnel Plot. **Fig C:** Funnel Plot with Trim and fill effect revealing missing studies. **Table A:** System and study based characteristics described in 226 studies compared to the 115 studies selected for the meta-regression and 86 studies for the hierarchical model respectively. **Table B:** Differences in Mortality, Length of Stay in the ICU and duration of Mechanical ventilation based on the World Bank economic development classification. The Median (range) of the Newcastle-Ottawa scale for different groups of studies. **Table C:** Differences in baseline characteristics based on the studies only describing unselected critically ill patients, studies describing patients undergoing mechanical ventilation, and studies describing patients under consideration or actually getting ECMO.
(DOCX)

S2 File PRSIMA Checklist. (DOC)

S3 File Data Sheet for H1N1 global variability manuscript. (XLSX)
