# Supplementary material for: Global Variability in Reported Mortality for Critical Illness during the 2009-10 Influenza A(H1N1) Pandemic: A Systematic Review and Meta-Regression to Guide Reporting of Outcomes during Disease Outbreaks
Source: PLoS One. 2016 May 12;11(5):e0155044. doi: 10.1371/journal.pone.0155044 (PMC4865181; doi:10.1371/journal.pone.0155044)
Supplement: S1 File — Fig A: Flowchart for subgroups of studies analyzed in the meta-regression. Fig B: Funnel Plot. Fig C: Funnel Plot with Trim and fill effect revealing missing studies. Table A: System and study based characteristics described in 226 studies compared to the 115 studies selected for the meta-regression and 86 studies for the hierarchical model respectively. Table B: Differences in Mortality, Length of Stay in the ICU and duration of Mechanical ventilation based on the World Bank economic development classification. The Median (range) of the Newcastle-Ottawa scale for different groups of studies. Table C: Differences in baseline characteristics based on the studies only describing unselected critically ill patients, studies describing patients undergoing mechanical ventilation, and studies describing patients under consideration or actually getting ECMO. (DOCX) [file pone.0155044.s001.docx]

**Supplement for Global variability in reported mortality for critical illness during the 2009-10 Influenza A(H1N1) Pandemic: A systematic review and meta-regression**

**Abhijit Duggal; Ruxandra Pinto; Gordon Rubenfeld; Robert A. Fowler**

**Search Strategy**

**MEDLINE search:**

**Influenza A(H1N1) Virus Search terms:**

1. exp Pandemics

2. exp Influenza, Human

3. exp Disease Outbreaks

4. exp Influenza A Virus, H1N1 Subtype

5. exp Influenza A Virus

**Critical Illness search terms:**

1. exp Critical Care

2. exp Intensive Care Units

3. exp Critical Illness

4. exp Intensive Care

5. exp Mechanical Ventilation

6. exp Artificial ventilation

7. exp Vasopressors

8. exp Inotropes

**EMBASE search:**

**Influenza A(H1N1) Virus Search terms:**

1. exp Influenza virus A H1N1/

2. exp Pandemic influenza/

**Critical Illness Search terms:**

1. exp Intensive care/ or exp intensive care unit/

2. exp Critical illness/

3. exp Critically ill patient/

4. exp Mechanical Ventilation

5. exp Artificial ventilation

6. exp Vasopressors

7. exp Inotropes

**LILACS and African Index Medicus search**

1. exp Influenza virus A H1N1/

2. exp Pandemic influenza/

**Definitions**

**Outbreaks, Epidemics and Pandemics**

A disease outbreak is defined as the occurrence of a new disease or the reporting of a higher number of new cases of a disease than would be normally expected in a defined geographical area or temporal period. [[1](#_ENREF_1)] Disease outbreaks can occur in restricted geographical areas, or can involve several countries. Similarly, outbreaks can last for anywhere from a few days to several years [[1](#_ENREF_1)]. An epidemic is an outbreak that affects a large population in a more expansive geographical area, usually over a relatively short period of time. [[2](#_ENREF_2)] An understanding of the usual prevalence of a disease is important before the determination of an epidemic is made. [[2](#_ENREF_2)] Propagation of epidemics is dependent on an adequate number of susceptible hosts to an infectious agent. Epidemics that spread over several countries or continents, usually affecting a large population are called Pandemics. [[3](#_ENREF_3)] Pandemics frequently present in multiple waves of infections, where the numbers of infections and deaths can present in well-separated temporal peaks with a separation time-scale of months.

**2009-10 Influenza A (H1N1) Pandemic**

The 2009-2010 Influenza A(H1N1) pandemic was declared due to infections caused by a then variant of the Influenza A virus, that originated from animal influenza viruses and was unrelated to recent human seasonal influenza A(H1N1) viruses. The first cases of disease associated with pandemic H1N1 virus were reported in April 2009 from Mexico and the Southwestern United States. [[4](#_ENREF_4), [5](#_ENREF_5)]The disease spread quickly through the rest of the world and by 11 June 2009, WHO had declared a pandemic phase 6 alert.[[6](#_ENREF_6)] The 2009 H1N1 variant of influenza was the first recognized Pandemic of the 21st Century.[[6](#_ENREF_6)]

**World Health Organization definitions of H1N1 Pandemic**

WHO and CDC developed specific case definitions for 2009 H1N1 influenza. [[6-8](#_ENREF_6)]

a. Confirmed H1N1: An individual with an acute febrile respiratory illness and laboratory-confirmed pandemic (H1N1) 2009 virus infection by one or more of the following tests: real-time (RT)-PCR or viral culture; viral culture; 4-fold rise in pandemic (H1N1) 2009 virus-specific neutralizing antibodies.

b. Probable: An individual with an acute febrile respiratory illness who is positive for influenza A by influenza RT-PCR, but is un-typeable by regents used to detect different strains; or, positive for influenza A by an influenza rapid test or an influenza immunofluorescence assay (IFA) and meets criteria for a suspected case.

c. Suspected: An individual with acute febrile respiratory illness with onset within 7 days of close contact with a person who is a confirmed case of influenza A (H1N1) virus infection, or within 7 days of travel to a community either locally or internationally where there are one or more confirmed influenza A (H1N1) cases, or resides in a community where there are one or more confirmed influenza A (H1N1) cases.

**Waves of the H1N1 pandemic**

The first wave of the 2009 pandemic in the North America began in March 2009 and peaked in late June and early July 2009. [[4](#_ENREF_4), [9](#_ENREF_9)] There were markedly fewer cases throughout August, and the second larger wave peaked in late October and, early November. The first wave in the Southern Hemisphere occurred from May 2009 till August 2009. Also while many countries (e.g. United States and Canada) experienced at least two waves of infections during the 2009 pandemic, other countries (e.g. China) experienced only a single predominant wave of infection.[[10](#_ENREF_10)]

**Critical Illness**

There is no single accepted definition of critical illness. However, patients with critical illness often (but not always) have high complexity of disease, associated with actual or a high risk of organ dysfunction. Critical illness syndromes can be difficult to diagnose, often have a short prodrome, and usually are associated with higher mortality than patients with similar spectra of comorbid conditions and acute presentations without critical illness.[[11](#_ENREF_11)] Disease syndromes such as septic shock, and organ dysfunction such as acute respiratory distress syndrome, and acute renal injury are closely associated with the development of critical illness.

**World Bank Economic Development**

The World Bank classifies the vast majority of the world’s countries into one of four broad categories based on the per capita income: low income economies, lower-middle income economies, upper-middle income economies and high income economies. [[12](#_ENREF_12)] The composition of these groupings is intended to reflect basic economic country conditions

**Geographic regions of the world**

Most studies evaluating the global burden of disease describe differences between populations at a country level. [[13](#_ENREF_13)] It is difficult to accurately compare such differences in critical illness because of the inherent differences in patients and resources in different countries.[[11](#_ENREF_11)] A number of studies have described these differences at the level of different regions and continents.[[13](#_ENREF_13)] For this manuscript we explored the differences in outcomes at the level of continents, and then based on geographical region of the included countries. We used geographical regions based on the World Bank classification as follows: North America (Canada and United States of America); Europe and Central Asia (Albania, Hungary, Romania, Armenia, Kazakhstan, Serbia, Azerbaijan, Kosovo, Tajikistan, Belarus, Kyrgyz Republic, Turkey, Bosnia and Herzegovina, Macedonia, FYR, Turkmenistan, Bulgaria, Moldova, Ukraine, Georgia, Montenegro, Uzbekistan); East Asia and Pacific (American Samoa, Malaysia, Samoa, Cambodia, Marshall Islands, Solomon Islands, China, Micronesia, Fed. Sts, Thailand, Fiji, Mongolia, Timor-Leste, Indonesia, Myanmar, Tuvalu, Kiribati, Palau, Tonga, Dem. Rep. Korea, Papua New Guinea, Vanuatu, Lao PDR, Philippines, Vietnam); South Asia (Afghanistan, India, Pakistan, Bangladesh, Maldives, Sri Lanka, Bhutan, Nepal); Middle East and North Africa(Algeria, Jordan, Tunisia, Djibouti, Lebanon, West Bank and Gaza, Egypt, Libya, Yemen, Iran, Morocco, Iraq, Syrian Arab Republic); Sub-Saharan Africa (Angola, Gambia, Rwanda, Benin, Ghana, São Tomé and Principe, Botswana, Guinea, Senegal, Burkina Faso, Guinea-Bissau, Seychelles, Burundi, Kenya, Sierra Leone, Cameroon, Lesotho, Somalia, Cabo Verde, Liberia, South Africa, Central African Republic, Madagascar, South Sudan, Chad, Malawi, Sudan, Comoros, Mali, Swaziland, Dem. Rep Congo, Mauritania, Tanzania, Congo, Mauritius, Togo, Côte d'Ivoire, Mozambique, Uganda, Eritrea, Namibia, Zambia, Ethiopia, Niger, Zimbabwe, Gabon, Nigeria); Latin America and the Caribbean (Argentina, Ecuador, Nicaragua, Belize, El Salvador, Panama, Bolivia, Grenada, Paraguay, Brazil, Guatemala, Peru, Colombia, Guyana, St. Lucia, Costa Rica, Haiti, St. Vincent and the Grenadines, Cuba, Honduras, Suriname, Dominica, Jamaica, Venezuela, RB, Dominican Republic, Mexico) and Australia and New Zealand

**Acute Respiratory Distress Syndrome (ARDS)**

We defined ARDS based on the Berlin definition. [[14](#_ENREF_14)] Even though this definition was formulated after the 2009 Influenza A (H1N1) pandemic, we decided to use this as it is the most appropriate definition for a diagnosis of ARDS. ARDS was defined as: I. Bilateral opacities, unexplained by nodules, atelectasis or effusion on either chest radiograph or CT scan; and II. New or worsening respiratory symptoms or a clinical insult associated with ARDS within 7 days of diagnosis; and III. Objective assessment of cardiac function with modalities such as echocardiography to exclude cardiogenic pulmonary edema and; IV. Hypoxemia, with a PaO2/FiO2 ≤300 mm Hg despite non-invasive or Invasive mechanical ventilation with a PEEP (Positive End Expiratory Pressure) or Continuous Positive Airway Pressure (CPAP)≥ 5 cm H2O.[[14](#_ENREF_14)]

**Mechanical Ventilation**

Mechanical ventilation is a method to mechanically assist spontaneous or absent breathing attempts. It is the use of positive pressure to force a predetermined mixture of air into the central airways and alveoli of the lungs. This positive pressure ventilation can be provided either invasively (with the means of an endotracheal tube) or non-invasively (with the use of nasal, or full face masks). For the purpose of this study we defined mechanical ventilation as the use of any device used to provide positive pressure ventilation to the patients. We defined non-invasive mechanical ventilation as the use of facemasks to provide non-Invasive positive pressure ventilation (NPPV), bilevel pressure ventilation, or continuous positive airway pressure (CPAP). Invasive mechanical ventilation was defined as the use of positive pressure ventilation with any conventional or non-conventional mode of mechanical ventilation with the means of an endotracheal tube (ETT)

**Rescue therapies**

Rescue therapies are defined as the use of adjunctive clinical strategies in patients with severe hypoxemia. Rescue therapies include the following therapeutic interventions (prone position ventilation, high-frequency oscillatory ventilation (HFOV), airway pressure release ventilation (APRV) and extracorporeal membrane oxygenation (ECMO).

**High-frequency oscillatory ventilation (HFOV)**

High-frequency oscillatory ventilation (HFOV) provides pressure oscillations around a relatively constant mean airway pressure at very high rates (3–15 breaths per second). As a result very small tidal volumes are achieved with active inspiration and expiration.[[15](#_ENREF_15)] Although commonly used as a rescue therapy in 2009-2010, with the publication of recent clinical trials demonstrating potential harm, HFOV is no longer widely recommended as a rescue strategy.

**Airway pressure release ventilation (APRV)**

Airway pressure release ventilation (APRV) is a form of pressure control intermittent mandatory ventilation (PC-IMV) typically used in the setting of ARDS and severe hypoxemia. During APRV, airway pressure is set at 2 levels, sometimes called for 2 time periods and effectively raises the mean airway pressure, recruits and helps to maintain open alveoli that can then participate in gas exchange. The effect on clinical outcomes of patients with ARDS is uncertain.[[16](#_ENREF_16)]

**Extracorporeal membrane oxygenation (ECMO)**

Extracorporeal membrane oxygenation uses degrees of cardiopulmonary bypass technology to provide gas exchange and to augment blood flow. In patients with severe hypoxemia this modality can increase oxygenation and ventilation while allowing a lung protective ventilation strategy with low tidal volume breaths. With the advent of new technology such as veno-venous circuits and smaller cannulas, the use of ECMO has gained more acceptance as a therapy in patients with ARDS. This trend was seen with the use of ECMO in patients with severe or refractory hypoxemia associated with ARDS during the H1N1 pandemic. [[17](#_ENREF_17)]

**Prone Position Ventilation**

Prone position ventilation is the use of invasive mechanical ventilation to patients in the prone (lying on the chest and abdomen as opposed to lying on the back) position.[[18](#_ENREF_18)] The use of this intervention has been associated with a significant risk reduction in mortality in one clinical trial.[[19](#_ENREF_19)]

**Sepsis /Severe Sepsis and Septic Shock**

Sepsis, severe sepsis and septic shock have been defined based on an international consensus statement developed by the Society for Critical Care Medicine (SCCM) Surviving Sepsis.[[20](#_ENREF_20)] Sepsis is defined as the presence (probable or documented) of infection together with systemic inflammatory manifestations. Severe sepsis is defined as sepsis plus sepsis-induced organ dysfunction. Septic shock was defined as sepsis-induced hypotension persisting despite adequate fluid resuscitation, which may be defined as infusion of 30 mL/kg of crystalloids bolus over 10-15 minutes.[[20](#_ENREF_20)]

**Vasoactive Medications**

Medications that induce vasoconstriction and thereby elevate mean arterial pressure (MAP) are called vasopressors. Inotropes are medications that increase cardiac contractility. [[21](#_ENREF_21), [22](#_ENREF_22)] Many drugs have both vasopressor and inotropic effects. For the purpose of our study we defined the use of the common vasopressors (e.g. norepinephrine, vasopressin, epinephrine, dopamine and phenylephrine) or Inotropes (e.g. dobutamine, milrinone) as vasoactive medication use.

**Acute Renal Failure**

Acute Renal failure is defined as the worsening of serum creatinine and glomerular filtration rate (GFR), a decrease in the urine output with a risk of progression to chronic renal insufficiency or failure. Recently acute renal failure has been defined based on the Risk, Injury, Failure, Loss, and End stage renal disease (RIFLE) Criteria. The changes in the serum creatinine, urine output and glomerular filtration rate (GFR) help in defining the severity of disease. Worsening kidney dysfunction is labeled as Risk, Injury, and Failure respectively. The RIFLE criterion uses short and long term outcomes to define Loss and ESRD. [[23](#_ENREF_23)]

**Severity of Illness scores:** These are scoring systems used in critically ill patients to assess the severity of disease and provide an estimate of in-hospital mortality. The estimate is based on collection of specific clinical and/or physiologic variables with different weighting. These severity scores are then used to calculate the probability of mortality among patients. Ideal scoring systems should be easy to collect, be well-calibrated, have a high level of discrimination and should be generalizable across various patient populations [[24](#_ENREF_24)] For the purpose of our study we have collected data on the following severity of illness scores.

APACHEII/III/IV: The Acute Physiologic and Chronic Health Evaluation (APACHE) scoring system is a severity score used to predict hospital mortality. Age, diagnosis at the time of admission, and numerous acute physiologic and chronic health variables are a part of the APACHE Score. [[24](#_ENREF_24), [25](#_ENREF_25)]

SAPSII/III: Simplified Acute Physiology score (SAPS) is a severity of disease classification system that describes the morbidity in patients based on 12 routine physiologic measurements. [[26](#_ENREF_26)]

SOFA: The Sequential Organ Failure Assessment (SOFA) uses simple measurements of six major organ functions to calculate a severity score. Serial measurements of this score are predictive of mortality in critically ill patients. [[27](#_ENREF_27)]

PRISM III: The PRISM III is a scoring system used to predict critical care outcomes for pediatric patients. It describes severity of illness or injury in this population. [[28](#_ENREF_28)]

**Co-Morbidities**

Presence of one or more medical conditions that existed in addition to the most significant condition (usually recorded as the "most responsible diagnosis" on hospital discharge abstracts) that caused a patient's stay in the hospital. The number of comorbid conditions is used to provide an indication of the health status (and is also used to help estimate the risk of death) of patients.

Heart Disease: Described as the presence of documented any coronary artery disease, congestive heart failure, congenital heart disease, valvular abnormalities and chronic arrhythmias

Lung Disease: Described as the presence of any asthma, interstitial lung disease, chronic obstructive pulmonary disease (COPD) including chronic bronchitis and emphysema; bronchiectasis, cystic fibrosis, pneumoconiosis and bronchopulmonary dysplasia (BPD)

Immunosuppression: Immunodeficiency related to use of immunosuppressive drugs (e.g. chemotherapy) or systemic steroids, Human Immunodeficiency Virus infection and Acquired Immune Deficiency Syndrome and autoimmune diseases resulting in systemic immunodeficiency.

Malignancy: Defined as the presence of any metastatic solid or hematological malignancy.

Obesity: We used the WHO definition of obesity for our study, defined as a Body Mass Index (BMI) of > 30 kg/m^2^. BMI is calculated as body weight in kilograms divided by the square of the height in meters (kg/m^2^).

Pregnancy: We defined pregnancy as a state for any female who was either pregnant or post-partum (within 6 weeks of delivery) at the time of H1N1 infection.

**Eligibility criteria for different sub-group of studies**

We anticipated that many early and potentially smaller studies would describe patients subsequently included in multicenter or national studies. To prevent non-independent reporting of patient characteristics and outcomes, we included studies only representing unique patient populations for the description of outcomes over different geographical or economic regions and specific ICU populations; however we included studies with potentially duplicated patients for description of outcomes over time, and for single versus multiple centers comparisons. One of the key statistical challenges therefore was to ensure that our estimates were not affected by the duplication of data due to multiple manuscripts describing the same patients. Therefore, we divided all the manuscripts based on the country of enrollment of the patients. We then further evaluated whether the manuscripts were a part of a national database, or not. If they were, we recognized them as being non-duplicate only if they were reporting on cases from different time periods of the pandemic. For studies that were performed in countries without a central data collection mechanism, we reviewed the information on the included medical centers reported in the manuscript, and a study was recognized as being a non-duplicate study only if the centers were different, or if the same centers reported outcomes at different time points. Different articles were used to describe the effect of system, study and patient based variables, so we have described our methodology for all these groups in details

**Time as a factor in the reporting of mortality:** For this analysis we excluded duplicate studies (both databases, and studies with overlapping patients or reporting a similar time period) and studies with only pediatric patients (as pediatric mortality was low and not comparable to adult patients).

**Geography and economic development as factors in the reporting of the mortality:** We collected the data on mortality from different countries; we excluded duplicate studies (from similar databases, or reporting on overlapping patients during similar time period). As there was significant heterogeneity in the severity of disease, occurrence of organ failure and the use of ICU specific therapies in studies from different geographical domains, we also identified the differences in mortality among “unselected” critically ill adults, to examine difference in the reporting of mortality in a homogenous group of studies at a global level and to obtain the most valid estimate of mortality among critically ill patients world-wide.

**Influence of specific ICU population on the reporting of mortality:** We excluded duplicate studies (any study that might have reported similar patients were screened, and the only studies describing patients over non-overlapping times for each country were included). We excluded studies reporting on only pediatric populations.

**Age as a factor in the reporting of mortality:** We report mortality from non-duplicate studies for pediatric, adult and both pediatric and adult cohorts.

**Influence of single center or multicenter studies on the reporting of mortality:** We excluded duplicate studies (any study that could have reported similar patients was screened, and only the study that reported on the most number of patients for the longest time period were included, specific group of patients reported at different times for each country were included).

**Influence of the number of patients in a study on the reporting of mortality:** We included all the studies that met our inclusion criteria

**Mortality in specific sub-groups of critically ill patients:** We selected non-duplicate studies in adults.

**S1 Fig: Flowchart for subgroups of studies analyzed in the meta-regression**

Studies included in qualitative synthesis
(n = 220)**$**

Studies evaluating time of enrollment

(n=107)**^+^**

Comparison of:

-Number of patients enrolled

(n=115)*

-Adults vs Pediatrics vs both

(n=131)^@^

-Single vs Multicenter

(n=115)*

Studies evaluating specific ICU population

(n=115)*

Studies evaluating geography/ economic development

(n=115)*

Studies $: [[29-242](#_ENREF_29)] [[243](#_ENREF_243)] [[244-246](#_ENREF_244)] [[247](#_ENREF_247)] [[248](#_ENREF_248), [249](#_ENREF_249)] [[250](#_ENREF_250)]

Studies *: [[29-33](#_ENREF_29), [36](#_ENREF_36), [38](#_ENREF_38), [41](#_ENREF_41), [42](#_ENREF_42), [46](#_ENREF_46), [48](#_ENREF_48), [49](#_ENREF_49), [51](#_ENREF_51), [54-58](#_ENREF_54), [64](#_ENREF_64), [69](#_ENREF_69), [71](#_ENREF_71), [73](#_ENREF_73), [76](#_ENREF_76), [77](#_ENREF_77), [79](#_ENREF_79), [81](#_ENREF_81), [83-85](#_ENREF_83), [87](#_ENREF_87), [94](#_ENREF_94), [97-101](#_ENREF_97), [103](#_ENREF_103), [104](#_ENREF_104), [107-109](#_ENREF_107), [111](#_ENREF_111), [112](#_ENREF_112), [116-118](#_ENREF_116), [120](#_ENREF_120), [121](#_ENREF_121), [123-125](#_ENREF_123), [128](#_ENREF_128), [134](#_ENREF_134), [136-138](#_ENREF_136), [140](#_ENREF_140), [141](#_ENREF_141), [143-145](#_ENREF_143), [147](#_ENREF_147), [150](#_ENREF_150), [154-156](#_ENREF_154), [158](#_ENREF_158), [159](#_ENREF_159), [165](#_ENREF_165), [167-169](#_ENREF_167), [171](#_ENREF_171), [173-175](#_ENREF_173), [179-182](#_ENREF_179), [186-188](#_ENREF_186), [192](#_ENREF_192), [194](#_ENREF_194), [197](#_ENREF_197), [199](#_ENREF_199), [201](#_ENREF_201), [202](#_ENREF_202), [204-206](#_ENREF_204), [208-211](#_ENREF_208), [213](#_ENREF_213), [214](#_ENREF_214), [217](#_ENREF_217), [220](#_ENREF_220), [221](#_ENREF_221), [224](#_ENREF_224), [226](#_ENREF_226), [228](#_ENREF_228), [230](#_ENREF_230), [231](#_ENREF_231), [234](#_ENREF_234), [236](#_ENREF_236), [242](#_ENREF_242)]

Studies ^@^:[[29-33](#_ENREF_29), [35-38](#_ENREF_35), [42](#_ENREF_42), [46](#_ENREF_46), [48-51](#_ENREF_48), [54-58](#_ENREF_54), [64](#_ENREF_64), [69](#_ENREF_69), [71](#_ENREF_71), [73](#_ENREF_73), [76](#_ENREF_76), [77](#_ENREF_77), [79-81](#_ENREF_79), [83-85](#_ENREF_83), [87](#_ENREF_87), [94](#_ENREF_94), [97-101](#_ENREF_97), [103](#_ENREF_103), [104](#_ENREF_104), [107-109](#_ENREF_107), [111](#_ENREF_111), [112](#_ENREF_112), [116-118](#_ENREF_116), [120](#_ENREF_120), [121](#_ENREF_121), [123-125](#_ENREF_123), [128](#_ENREF_128), [134](#_ENREF_134), [136-138](#_ENREF_136), [140](#_ENREF_140), [141](#_ENREF_141), [143-145](#_ENREF_143), [147](#_ENREF_147), [149](#_ENREF_149), [154-156](#_ENREF_154), [158](#_ENREF_158), [159](#_ENREF_159), [165](#_ENREF_165), [167-169](#_ENREF_167), [172-175](#_ENREF_172), [179-182](#_ENREF_179), [186-188](#_ENREF_186), [192](#_ENREF_192), [194](#_ENREF_194), [197](#_ENREF_197), [199](#_ENREF_199), [201](#_ENREF_201), [202](#_ENREF_202), [204-206](#_ENREF_204), [208-211](#_ENREF_208), [213](#_ENREF_213), [214](#_ENREF_214), [217](#_ENREF_217), [220](#_ENREF_220), [221](#_ENREF_221), [223](#_ENREF_223), [226](#_ENREF_226), [228](#_ENREF_228), [230](#_ENREF_230), [231](#_ENREF_231), [234](#_ENREF_234), [236](#_ENREF_236), [242](#_ENREF_242)] [[59](#_ENREF_59), [72](#_ENREF_72), [90](#_ENREF_90), [93](#_ENREF_93), [122](#_ENREF_122), [126](#_ENREF_126), [129](#_ENREF_129), [131](#_ENREF_131), [142](#_ENREF_142), [151](#_ENREF_151), [164](#_ENREF_164), [184](#_ENREF_184), [216](#_ENREF_216), [219](#_ENREF_219), [227](#_ENREF_227), [240](#_ENREF_240), [241](#_ENREF_241)] [[244](#_ENREF_244), [250](#_ENREF_250)]

Studies+: [[29-33](#_ENREF_29), [36](#_ENREF_36), [38](#_ENREF_38), [42](#_ENREF_42), [49](#_ENREF_49), [51](#_ENREF_51), [54-58](#_ENREF_54), [64](#_ENREF_64), [71](#_ENREF_71), [73](#_ENREF_73), [76](#_ENREF_76), [77](#_ENREF_77), [79-81](#_ENREF_79), [83-85](#_ENREF_83), [87](#_ENREF_87), [94](#_ENREF_94), [98-101](#_ENREF_98), [103](#_ENREF_103), [104](#_ENREF_104), [107-109](#_ENREF_107), [111](#_ENREF_111), [112](#_ENREF_112), [116](#_ENREF_116), [118](#_ENREF_118), [120](#_ENREF_120), [121](#_ENREF_121), [123-125](#_ENREF_123), [134](#_ENREF_134), [136-138](#_ENREF_136), [140](#_ENREF_140), [141](#_ENREF_141), [143-145](#_ENREF_143), [147](#_ENREF_147), [150](#_ENREF_150), [152](#_ENREF_152), [154-156](#_ENREF_154), [158](#_ENREF_158), [159](#_ENREF_159), [165](#_ENREF_165), [167-169](#_ENREF_167), [171](#_ENREF_171), [173-175](#_ENREF_173), [179-182](#_ENREF_179), [186-188](#_ENREF_186), [192](#_ENREF_192), [194](#_ENREF_194), [197](#_ENREF_197), [199](#_ENREF_199), [201](#_ENREF_201), [202](#_ENREF_202), [204-206](#_ENREF_204), [208-211](#_ENREF_208), [213](#_ENREF_213), [214](#_ENREF_214), [217](#_ENREF_217), [220](#_ENREF_220), [221](#_ENREF_221), [223](#_ENREF_223), [228](#_ENREF_228), [230](#_ENREF_230), [231](#_ENREF_231), [234](#_ENREF_234), [236](#_ENREF_236), [239](#_ENREF_239), [242](#_ENREF_242)]

**Statistical Analysis**

**Subgroup analysis and Meta-Regression**

Subgroup analyses and meta-regression are methods to investigate differences between studies. Statistical significance of the results within separate subgroup analyses should not be compared and we have to be mindful of possible bias through confounding by other study-level characteristics when we consider sub-group analyses. For patient and intervention characteristics, differences in subgroups that are observed within studies are more reliable than analyses of subsets of studies. [[251](#_ENREF_251)] Meta-regression is an extension to subgroup analyses that allows the effect of continuous, as well as categorical, characteristics to be investigated, and in principle allows the effects of multiple factors to be investigated simultaneously. Meta-regression should generally not be considered when there are fewer than ten studies in a meta-analysis. [[251](#_ENREF_251)]

we explored clinical heterogeneity by establishing subgroups of studies according to distinct patient populations and conducted subgroup analyses based on different variables extracted from the studies, including specific pandemic time periods (first wave, second wave, prolonged enrollment), geographical region (country, region, continent, World Bank economic development status), study population characteristics (unselected patients, mechanically ventilated), co-morbidities (pregnancy or post-partum), specific illnesses (ARDS, acute kidney injury) and ICU specific interventions such as receipt of rescue oxygenation therapy (ECMO, HFOV). Different subgroups are analyzed as follows.

**Time as a factor in the reporting of mortality:** We divided the pandemic into distinct time-points (based on the enrollment of the patients to the individual studies) and described the mortality associated with Wave I (April 1, 2009 to August 31 2009), Wave II (September 1 2009 to January 31 2010), and for patients enrolled from February 1, 2010. We anticipated a significant overlap of enrollment between these distinct waves of the pandemic. Due to this we also reported on the mortality associated with studies enrolling for between 5 to 9 months of the pandemic and for studies enrolling for more than 9 months of the pandemic (these studies were assessed together regardless of the time period of enrollment). As one of the main hypothesis of our study was to investigate whether early reporting of pandemics was associated with a difference in reported mortality we further performed a paired analysis for all the counties that reported during Wave I of the pandemic with studies from the same countries that enrolled for longer than 9 months. These results were presented as a risk difference, which is defined as the difference between the observed risks in two groups under study. The risk difference describes the estimated difference in the probability of experiencing an event.

**Geography and economic development as factors in the reporting of the mortality:** We reported on mortality at three geographical levels: 1. World Bank region; 2. Continent; and, 3. Hemisphere. We also report the mortality using the same cohort of studies as described above after using the World Bank categorization for high income, upper and lower middle income and low income economy countries.

**Influence of specific ICU population on the reporting of mortality:** We divided the studies into three distinct categories which we believe signified differing severity of disease in the cohorts that were being evaluated, on the basis of mortality estimates from non-H1N1 populations: unselected critically ill patients**;** mechanically ventilated patients**;** and patients undergoing non-conventional mechanical ventilation. We compared the mortality for the three groups, to determine the influence of severity of illness. We also summarized the differences in the duration of mechanical ventilation and length of stay in the ICU for each sub-group.

**Age as a factor in the reporting of mortality:** Because of the heterogeneity among the pediatric group, we did not include pediatric studies for our comparative analyses and we report a comparison of studies with only adults with studies that describe patients of all ages.

**Influence of single center or multicenter studies on the reporting of mortality:** We compared reported mortality from multicenter studies compared to single center studies.

**Influence of the number of patients in a study on the reporting of mortality:** Based on a priori discussion and review of various cohort studies reporting on the Influenza A (H1N1) pandemic we divided the studies into 6 sub-groups. These were based on the number of patients described in each manuscript: 10 or less; 11 to 25; 26-100; 101-250; and >250. We then compared the difference in cumulative mortality in all these sub-groups.

**Mortality in specific sub-groups of critically ill patients:** We reported the mortality in sub-groups of specific patients. We report mortality associated with co-morbidities or co-presenting conditions (e.g. pregnancy). We also report on studies of that included patients based upon their receipt of specific therapies such as mechanical ventilation, ECMO, HFOV; and, common organ system failures (ARDS, acute kidney injury)

**S1 Table: System and study based characteristics described in 219 studies compared to the 113 studies selected for the meta-regression and 86 studies for the hierarchical model respectively.**

| **Study Characteristics** | **All Studies**  **(n=219)** | **Studies for Meta-regression**  **(n=115)** |
| --- | --- | --- |
| Period of Enrollment  April 2009- August 2009  September 2009-January 2010  February 2010 till end of pandemic  Studies enrolling through different waves of the Pandemic | 50 (22%)  31 (14%)  3 (1%)  144 (63%) | 21 (18%)  26 (23%)  1 (1%)  66 (58%) |
| Multicenter Studies | 109 (49%) | 46 (40%) |
| Study size (number of patients)  5-10  11-25  26-100  101-250  >250 | 35 (16%)  79 (35%)  68 (30%)  22 (10%)  21 (9%) | 23 (20%)  46 (40%)  30 (26%)  6 (5%)  10 (9%) |
| Studies with only adult patients | 140 (63%) | 81 (72%) |
| Studies describing unselected critically ill patients | 155(69%) | 71 (62%) |
| Studies describing specific subgroups  ARDS  Acute Kidney Injury  Pregnant critically ill  Mechanical Ventilation  ECMO | 59 (26%)  10 (4%)  8 (4%)  63 (28%)  20 (9%) | 37 (32%)  4 (4%)  3 (3%)  40 (35%)  8 (7%) |
| **Study geographical region** |  |  |
| Americas  North America*  Latin America and Caribbean^#^  Europe  Western Europe  Eastern Europe  Asia  Middle East  South Asia  East Asia and Pacific  Africa  North Africa  Sub-Saharan Africa  Australia/New Zealand | 43 (19%)  26 (11%)  69 (31%)  11 (5%)  12 (5%)  12 (5%)  32 (14%)  3 (1%)  3 (1%)  16 (7%) | 12 (11%)  14 (13%)  40 (35%)  10(9%)  6 (5%)  8 (7%)  17 (15%)  3 (3%)  3 (3%)  2 (2%) |
| Study country economic status of the country  High Income Economy  Upper Middle Income Economy  Lower Middle Income Economy | 161 (71%)  50 (22%)  13 (7%) | 75 (65%)  31 (27%)  9 (8%) |

Values are numbers (percentages) unless stated otherwise. We describe the system based, temporal and geographical characteristics of countries included in our systematic review. We also describe similar variables for studies included in our meta-regression and our hierarchical model. This table shows that at each level the relative distribution of the variables remained constant throughout the reported studies.

**Quality of Included studies**

**Risk of Bias and quality of evidence assessment**

The Newcastle-Ottawa Scale scores for the risk of bias ranged from 4 to 9 out of a maximum of 9 with a median of 7 across studies. Most of the studies were considered to be of high quality. As we were not comparing two distinct groups of patients we evaluated the risk for under- or over-reporting of mortality based on the three domains of the scale. We defined the risk as being high for studies with a score of 6 or lower.

**S2 Table: The Median (range) of the Newcastle-Ottawa scale for different groups of studies**

|  | **Overall Score** | **Selection of study groups** | **Comparability of groups** | **Ascertainment of exposure/ disease** |
| --- | --- | --- | --- | --- |
| **All studies** | **7** | **3** | **2** | **3** |
| **Based on period of Enrollment** |  |  |  |  |
| **Wave 1**  **Wave 2**  **Prolonged** | **7**  **8**  **7** | **3**  **3**  **3** | **2**  **2**  **2** | **3**  **3**  **3** |
| **Geographical Region** |  |  |  |  |
| **North America**  **Eastern Europe**  **Western Europe**  **Latin America and Caribbean**  **Australia/ New Zealand**  **East Asia**  **South Asia**  **Mid East and North Africa**  **Sub-Saharan Africa** | **8**  **7.5**  **7**  **7**  **7**  **7**  **8**  **8**  **6** | **3**  **3**  **2**  **3**  **3**  **3**  **3**  **3**  **2** | **2**  **2**  **2**  **2**  **1.5**  **1.5**  **2**  **2**  **1** | **3**  **3**  **3**  **3**  **3**  **3**  **3**  **3**  **3** |
| **Non-Selected Critically ill patients** | **8** | **3** | **2** | **3** |

Newcastle-Ottawa Scale describing the quality of the studies based on different subgroups. We describe the quality of the studies based on the time of enrollment, the geographical regions, and studies just describing non-selected critically ill patients. Most studies were considered to be of high quality based on our scoring criterion (decided *a priori*)

**S2 Fig: Funnel Plot**


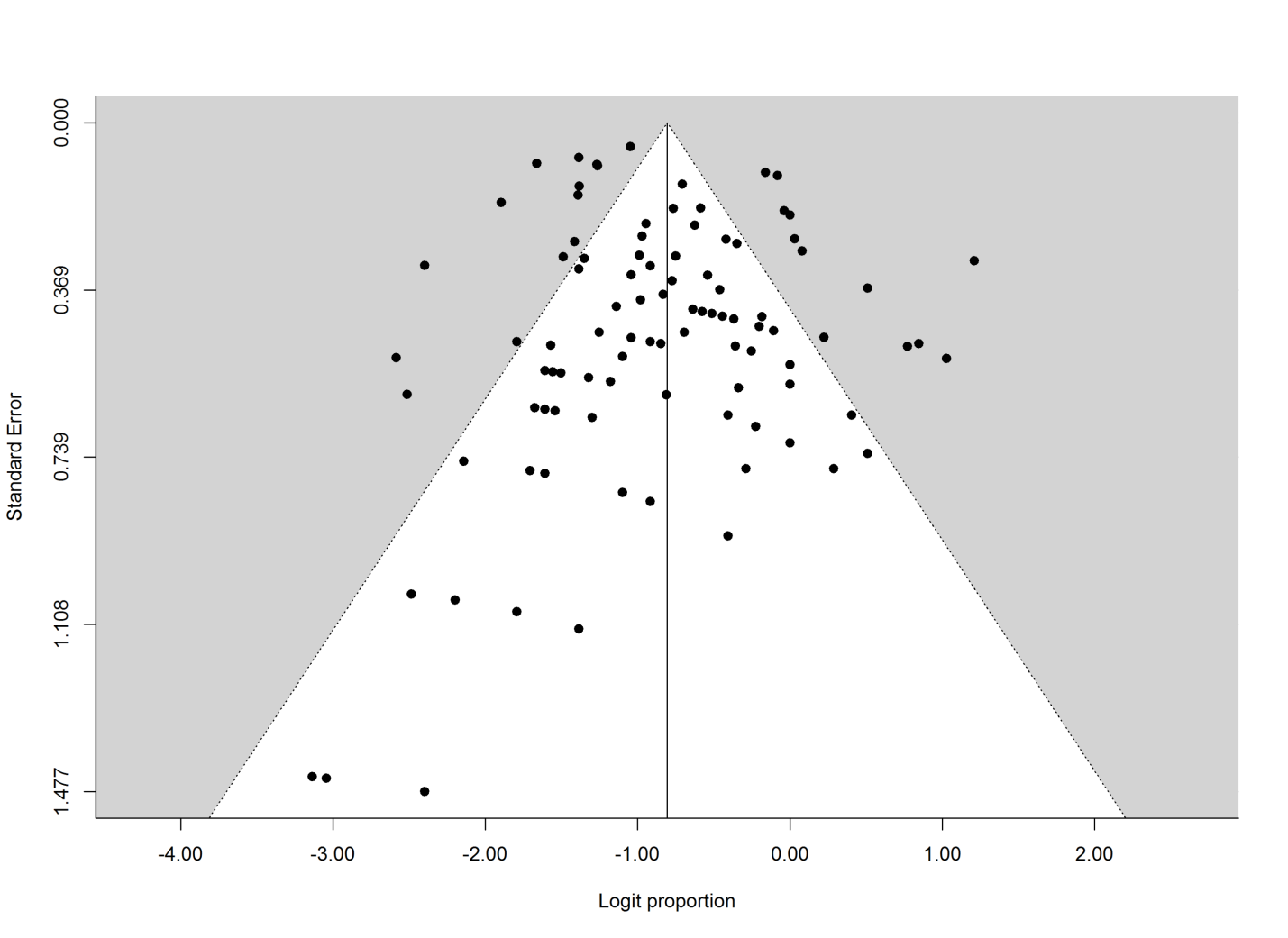


Funnel plot examining the risk of publication bias based on the logit proportion of mortality. There is a relative paucity of small studies with a large difference in mortality. Also there are only a few small studies with a small difference in mortality. These represent a specific group (pregnant females) with a very low mortality associated with Influenza A (H1N1) pandemic. There were only a few small studies with a small difference in mortality. These represent a specific group (pregnant females) with a very low mortality associated with Influenza A (H1N1) pandemic.

**S3 Fig: Funnel Plot with Trim and fill effect revealing missing studies**


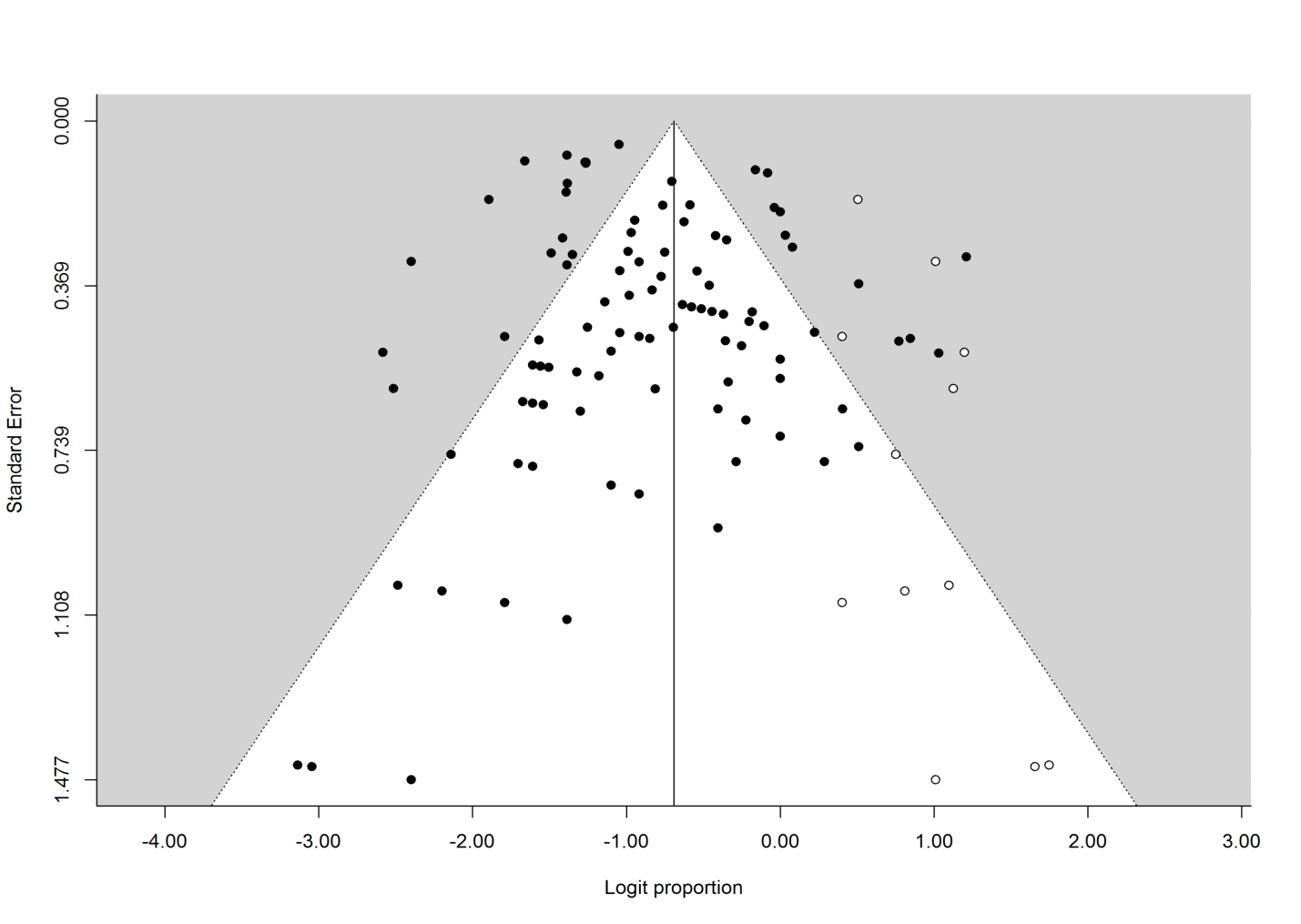


The black dots represent the individual studies with a distribution of the logit of reported mortality. The empty dots represent the potentially missing studies. We then used a trim and fill effect on the random effects model and estimated that data from 15 studies was missing. All these studies had a large difference in mortality but they were a mix of both small and large studies

**S2 Table: Differences in Mortality, Length of Stay in the ICU and duration of Mechanical ventilation based on the World Bank economic development classification**.

| **World Bank Economic development status** | **High income economy** | | **Upper middle income economy** | | **Lower middle income economy** | |
| --- | --- | --- | --- | --- | --- | --- |
| Short-term mortality | N |  | N |  | N |  |
|  | *161* | 24% | *50* | 35% | *13* | 52% |
| Duration of Mechanical Ventilation, days | *53* | 11 (8-16) | *13* | 9 (8-10) | *6* | 8 (6-10) |
| Length of Stay in ICU, days | *80* | 12 (8-20) | *14* | 10 (7-12) | *4* | 10 (7-11) |

Variables are described as median (interquartile range) unless stated otherwise. N Denotes the number of studies that reported the specific outcomes. ICU: Intensive Care Unit

**S3 Table**: **Differences in baseline characteristics based on the studies only describing unselected critically ill patients, studies describing patients undergoing mechanical ventilation, and studies describing patients under consideration or actually getting ECMO**

| **Characteristics** | **Unselected Critically ill**  **(n=155)** | | **Mechanical Ventilation**  **(n=46)** | | **Extracorporeal membrane Oxygenation**  **(n=20)** | |
| --- | --- | --- | --- | --- | --- | --- |
|  | n |  | n |  | n |  |
| Age | *122* | 41 (30-45) | *39* | 41 (35-46) | *14* | 36 (32-40) |
| Females | *119* | 47% | *38* | 50% | *16* | 51% |
| APACHE II | *60* | 18 (14-21) | *20* | 18 (16-21) | *6* | 18 (17-19) |
| Lung Disease | *107* | 28% | *25* | 23% | *10* | 14% |
| Obesity | *69* | 26% | *23* | 27% | *7* | 40% |
| Pregnancy | *71* | 9% | *19* | 9% | *12* | 23% |
| ARDS | *76* | 72% | *37* | 100% | *14* | 100% |
| Acute Renal Failure | *37* | 32% | *9* | 50% | *2* | 49% |
| Renal Replacement Therapy | *44* | 15% | *13* | 17% | *6* | 25% |
| Need for Inotropes | *65* | 43% | *24* | 55% | *8* | 65% |
| Antivirals | *60* | 99% | *26* | 100% | *5* | 100% |
| Antibiotics | *36* | 98% | *12* | 100% | *1* | 100% |
| Corticosteroids | *45* | 46% | *17* | 50% | *8* | 42% |
| Duration of Mechanical ventilation | *46* | 9 (7-11) | *18* | 12(9-19) | *8* | 22 (11-27) |
| ICU length of stay | *70* | 9 (7-12) | *20* | 12(10-20) | *7* | 22 (18-33) |
| Short term Mortality* | *155* | 25% | *47* | 36% | *20* | 32% |

The patient characteristics, co-morbidities and ICU specific interventions were similar in these sub-groups. Patients who underwent ECMO had longer duration of mechanical ventilation and length of stay in the ICU when compared to mechanically ventilated patients. Patients undergoing ECMO had a lower mortality that patients undergoing mechanical ventilation.

**List of Abbreviations**:

ARDS: Acute Respiratory Distress Syndrome; AKI: Acute Kidney Injury; AIDS: Acquired Immunodeficiency syndrome; CDC: Centers for Disease Control and Prevention; CI: Confidence Interval; CPAP: Continuous positive airway pressure;ECMO: Extracorporeal Membrane Oxygenation; ESRD: end stage renal disease; ETT: Endotracheal tube; FiO2: Fraction of inhaled oxygen; GFR: glomerular filtration rate; HFOV: High frequency oscillatory ventilation; HIV: Human immunodeficiency virus; ICU: Intensive Care Unit; IQR: interquartile range; MAP: mean arterial pressure; MeSH: medical subject headings; MERS: Middle East Respiratory Syndrome; NOS: Newcastle-Ottawa Scale;NPPV: Non-invasive positive pressure ventilation; PaO2: Partial Pressure of Oxygen; PEEP: Positive end expiratory pressure; PRISMA: Preferred reporting items for systematic reviews and meta-analyses;RIFLE: Risk, Injury, Failure, Loss, and End-stage renal disease; SARS: Severe Acute Respiratory Syndrome; SBP: systolic blood pressure; SCCM: Society for critical care medicine; SD: standard Deviation; WBC: white blood cell; WHO: World Health Organization.

**References:**

1. Organization WH. Disease Outbreaks 2015 [cited 2015 February 1st]. Available from: <http://www.who.int/topics/disease_outbreaks/en/>.

2. Prevention CfDCa. Epidmeic Disease Occurance 2012 [updated May 18, 2012; cited 2012]. Available from: <http://www.cdc.gov/ophss/csels/dsepd/ss1978/lesson1/section11.html>.

3. Control ECfDpa. Definition of a Pandemic 2012. Available from: <http://www.ecdc.europa.eu/en/healthtopics/pandemic_preparedness/basic_facts/Pages/definition_of_pandemic.aspx>.

4. Jain S, Kamimoto L, Bramley AM, Schmitz AM, Benoit SR, Louie J, et al. Hospitalized patients with 2009 H1N1 influenza in the United States, April-June 2009. N Engl J Med. 2009;361(20):1935-44. Epub 2009/10/10. doi: 10.1056/NEJMoa0906695. PubMed PMID: 19815859.

5. Dominguez-Cherit G, Lapinsky SE, Macias AE, Pinto R, Espinosa-Perez L, de la Torre A, et al. Critically Ill patients with 2009 influenza A(H1N1) in Mexico. Jama. 2009;302(17):1880-7. Epub 2009/10/14. doi: 10.1001/jama.2009.1536. PubMed PMID: 19822626.

6. Organization WH. Human Infection with Pandemic (H1N1) 2009 virus 2009 [December 20th,2014]. Available from: <http://www.who.int/csr/disease/swineflu/WHO_case_definition_swine_flu_2009_04_29.pdf>.

7. Chen SY, Chen YC, Chiang WC, Kung HC, King CC, Lai MS, et al. Field performance of clinical case definitions for influenza screening during the 2009 pandemic. Am J Emerg Med. 2012;30(9):1796-803. Epub 2012/05/29. doi: 10.1016/j.ajem.2012.02.016. PubMed PMID: 22633701.

8. Mahony AA, Cheng AC, Olsen KL, Aboltins CA, Black JF, Johnson PD, et al. Diagnosing swine flu: the inaccuracy of case definitions during the 2009 pandemic, an attempt at refinement, and the implications for future planning. Influenza Other Respir Viruses. 2013;7(3):403-9. Epub 2012/06/21. doi: 10.1111/j.1750-2659.2012.00398.x. PubMed PMID: 22712880.

9. Kumar A, Zarychanski R, Pinto R, Cook DJ, Marshall J, Lacroix J, et al. Critically ill patients with 2009 influenza A(H1N1) infection in Canada. Jama. 2009;302(17):1872-9. Epub 2009/10/14. doi: 10.1001/jama.2009.1496. PubMed PMID: 19822627.

10. Yu H, Feng Z, Uyeki TM, Liao Q, Zhou L, Feng L, et al. Risk factors for severe illness with 2009 pandemic influenza A (H1N1) virus infection in China. Clin Infect Dis. 2011;52(4):457-65. Epub 2011/01/12. doi: 10.1093/cid/ciq144. PubMed PMID: 21220768; PubMed Central PMCID: PMC3060897.

11. Adhikari NK, Rubenfeld GD. Worldwide demand for critical care. Curr Opin Crit Care. 2011;17(6):620-5. Epub 2011/11/10. doi: 10.1097/MCC.0b013e32834cd39c. PubMed PMID: 22067878.

12. Bank W. Country and lending groups: The World Bank 2014 [cited 2014 30th March]. Available from: <http://data.worldbank.org/about/country-and-lending-groups>.

13. Vincent JL, Marshall JC, Namendys-Silva SA, Francois B, Martin-Loeches I, Lipman J, et al. Assessment of the worldwide burden of critical illness: the Intensive Care Over Nations (ICON) audit. Lancet Respir Med. 2014;2(5):380-6. Epub 2014/04/18. doi: 10.1016/S2213-2600(14)70061-X. PubMed PMID: 24740011.

14. Ranieri VM, Rubenfeld GD, Thompson BT, Ferguson ND, Caldwell E, Fan E, et al. Acute respiratory distress syndrome: the Berlin Definition. Jama. 2012;307(23):2526-33. Epub 2012/07/17. doi: 10.1001/jama.2012.5669. PubMed PMID: 22797452.

15. Chan KP, Stewart TE, Mehta S. High-frequency oscillatory ventilation for adult patients with ARDS. Chest. 2007;131(6):1907-16. Epub 2007/06/15. doi: 10.1378/chest.06-1549. PubMed PMID: 17565024.

16. Habashi NM. Other approaches to open-lung ventilation: airway pressure release ventilation. Crit Care Med. 2005;33(3 Suppl):S228-40. Epub 2005/03/09. PubMed PMID: 15753733.

17. Abrams D, Brodie D. Extracorporeal Circulatory Approaches to Treat Acute Respiratory Distress Syndrome. Clin Chest Med. 2014;35(4):765-79. Epub 2014/12/03. doi: 10.1016/j.ccm.2014.08.013. PubMed PMID: 25453424.

18. Gattinoni L, Taccone P, Carlesso E, Marini JJ. Prone position in acute respiratory distress syndrome. Rationale, indications, and limits. Am J Respir Crit Care Med. 2013;188(11):1286-93. Epub 2013/10/19. doi: 10.1164/rccm.201308-1532CI. PubMed PMID: 24134414.

19. Guerin C, Reignier J, Richard JC, Beuret P, Gacouin A, Boulain T, et al. Prone positioning in severe acute respiratory distress syndrome. N Engl J Med. 2013;368(23):2159-68. Epub 2013/05/22. doi: 10.1056/NEJMoa1214103. PubMed PMID: 23688302.

20. Dellinger RP, Levy MM, Rhodes A, Annane D, Gerlach H, Opal SM, et al. Surviving sepsis campaign: international guidelines for management of severe sepsis and septic shock: 2012. Crit Care Med. 2013;41(2):580-637. Epub 2013/01/29. doi: 10.1097/CCM.0b013e31827e83af. PubMed PMID: 23353941.

21. Overgaard CB, Dzavik V. Inotropes and vasopressors: review of physiology and clinical use in cardiovascular disease. Circulation. 2008;118(10):1047-56. Epub 2008/09/04. doi: 10.1161/CIRCULATIONAHA.107.728840. PubMed PMID: 18765387.

22. Bracht H, Calzia E, Georgieff M, Singer J, Radermacher P, Russell JA. Inotropes and vasopressors: more than haemodynamics! Br J Pharmacol. 2012;165(7):2009-11. Epub 2011/11/15. doi: 10.1111/j.1476-5381.2011.01776.x. PubMed PMID: 22074274; PubMed Central PMCID: PMC3413839.

23. Bellomo R, Kellum JA, Ronco C. Defining and classifying acute renal failure: from advocacy to consensus and validation of the RIFLE criteria. Intensive Care Med. 2007;33(3):409-13. Epub 2006/12/14. doi: 10.1007/s00134-006-0478-x. PubMed PMID: 17165018.

24. Breslow MJ, Badawi O. Severity scoring in the critically ill: part 1--interpretation and accuracy of outcome prediction scoring systems. Chest. 2012;141(1):245-52. Epub 2012/01/05. doi: 10.1378/chest.11-0330. PubMed PMID: 22215834.

25. Knaus WA, Draper EA, Wagner DP, Zimmerman JE. APACHE II: a severity of disease classification system. Crit Care Med. 1985;13(10):818-29. Epub 1985/10/01. PubMed PMID: 3928249.

26. Apolone G, Bertolini G, D'Amico R, Iapichino G, Cattaneo A, De Salvo G, et al. The performance of SAPS II in a cohort of patients admitted to 99 Italian ICUs: results from GiViTI. Gruppo Italiano per la Valutazione degli interventi in Terapia Intensiva. Intensive Care Med. 1996;22(12):1368-78. Epub 1996/12/01. PubMed PMID: 8986488.

27. Vincent JL, Moreno R, Takala J, Willatts S, De Mendonca A, Bruining H, et al. The SOFA (Sepsis-related Organ Failure Assessment) score to describe organ dysfunction/failure. On behalf of the Working Group on Sepsis-Related Problems of the European Society of Intensive Care Medicine. Intensive Care Med. 1996;22(7):707-10. Epub 1996/07/01. PubMed PMID: 8844239.

28. Pollack MM, Patel KM, Ruttimann UE. The Pediatric Risk of Mortality III--Acute Physiology Score (PRISM III-APS): a method of assessing physiologic instability for pediatric intensive care unit patients. J Pediatr. 1997;131(4):575-81. Epub 1997/12/05. PubMed PMID: 9386662.

29. Abdulkader RCRM, Ho YL, de Sousa Santos S, Caires R, Arantes MF, Andrade L. Characteristics of acute kidney injury in patients infected with the 2009 influenza A (H1N1) virus. Clinical Journal of The American Society of Nephrology: CJASN. 2010;5(11):1916-21. PubMed PMID: 20671226; PubMed Central PMCID: PMCPMC3001779.

30. Abouchacra S, Chaaban A, Gebran N, Hassan M, Paul A, Eid HA, et al. Acute kidney injury in patients with H1N1 admitted to ICU: Incidence, patient and renal outcomes. A multi-center perspective. Anaesthesia, Pain and Intensive Care. 2010;14(1):13-6. PubMed PMID: 2010467413.

31. Adeniji KA, Cusack R. The Simple Triage Scoring System (STSS) successfully predicts mortality and critical care resource utilization in H1N1 pandemic flu: a retrospective analysis. Critical Care (London, England). 2011;15(1):R39. PubMed PMID: 21269458; PubMed Central PMCID: PMCPMC3221968.

32. Adiguzel N, Karakurt Z, Kalamanoglu Balci M, Acarturk E, Gungor G, Yazicioglu Mocin O, et al. Influenza A (H1N1) virus pneumonia in intensive care unit. Tuberkuloz ve Toraks. 2010;58(3):278-85. PubMed PMID: 21038138.

33. Adlhoch C, Wadl M, Behnke M, Pena Diaz LA, Clausmeyer J, Eckmanns T. Pandemic influenza A(H1)pdm09 in hospitals and intensive care units - results from a new hospital surveillance, Germany 2009/2010. Influenza & Other Respiratory Viruses. 2012;6(6):e162-8. PubMed PMID: 22788851.

34. Agarwal PP, Cinti S, Kazerooni EA. Chest radiographic and CT findings in novel swine-origin influenza A (H1N1) virus (S-OIV) infection. AJR American Journal of Roentgenology. 2009;193(6):1488-93. PubMed PMID: 19933638.

35. Al Subaie SS, Al Saadi MA. Features associated with severe disease in hospitalized children with 2009 influenza A (H1N1) infection at a university hospital in Riyadh, Saudi Arabia. Annals of Saudi Medicine. 2012;32(1):53-8. PubMed PMID: 22156640.

36. Al-Lawati J, Al-Tamtami N, Al-Qasmi A, Al-Jardani A, Al-Abri S, Al Busaidy S. Hospitalised patients with Influenza A (H1N1) in the Royal Hospital, Oman: Experience of a tertiary care hospital, July-December 2009. Sultan Qaboos Univ Med J. 2010;10(3):326-34. Epub 2011/04/22. PubMed PMID: 21509252; PubMed Central PMCID: PMC3074727.

37. Altmann M, Fiebig L, Soyka J, von Kries R, Dehnert M, Haas W. Severe cases of pandemic (H1N1) 2009 in children, Germany. Emerg Infect Dis. 2011;17(2):186-92. PubMed PMID: 21291587; PubMed Central PMCID: PMCPMC3204773.

38. Anand R, Gupta A, Wadhawan S, Bhadoria P. Management of swine-flu patients in the intensive care unit: Our experience. Journal of Anaesthesiology Clinical Pharmacology. 2012;28(1):51-5. PubMed PMID: 2012120697.

39. Investigators AI, Webb SAR, Pettila V, Seppelt I, Bellomo R, Bailey M, et al. Critical care services and 2009 H1N1 influenza in Australia and New Zealand. New England Journal of Medicine. 2009;361(20):1925-34. PubMed PMID: 19815860.

40. Australia, New Zealand Extracorporeal Membrane Oxygenation Influenza I, Davies A, Jones D, Bailey M, Beca J, et al. Extracorporeal Membrane Oxygenation for 2009 Influenza A(H1N1) Acute Respiratory Distress Syndrome. Jama. 2009;302(17):1888-95. PubMed PMID: 19822628.

41. Investigators AI, Australasian Maternity Outcomes Surveillance S. Critical illness due to 2009 A/H1N1 influenza in pregnant and postpartum women: population based cohort study. Bmj. 2010;340:c1279. PubMed PMID: 20299694; PubMed Central PMCID: PMCPMC2841744.

42. Investigators AI, Webb SAR, Aubron C, Bailey M, Bellomo R, Howe B, et al. Critical care services and the H1N1 (2009) influenza epidemic in Australia and New Zealand in 2010: the impact of the second winter epidemic. Critical Care (London, England). 2011;15(3):R143. PubMed PMID: 21658233; PubMed Central PMCID: PMCPMC3219015.

43. Aquino-Esperanza J, Rodriguez PO, Boughen S, Raimondi A, Attie S, Maskin P, et al. [Severe respiratory disease in an intensive care unit during influenza A(H1N1) 2009 pandemia]. Medicina. 2010;70(5):401-7. PubMed PMID: 20920955.

44. Ariano RE, Sitar DS, Zelenitsky SA, Zarychanski R, Pisipati A, Ahern S, et al. Enteric absorption and pharmacokinetics of oseltamivir in critically ill patients with pandemic (H1N1) influenza. CMAJ Canadian Medical Association Journal. 2010;182(4):357-63. PubMed PMID: 20159892; PubMed Central PMCID: PMCPMC2831695.

45. Bagdure D, Curtis DJ, Dobyns E, Glode MP, Dominguez SR. Hospitalized children with 2009 pandemic influenza A (H1N1): comparison to seasonal influenza and risk factors for admission to the ICU. PLoS ONE [Electronic Resource]. 2010;5(12):e15173. PubMed PMID: 21179517; PubMed Central PMCID: PMCPMC3002273.

46. Bagnulo H, Soca A, Buroni M, Limongi G, Echavarria E, Noveri S, et al. Clinical profile and outcome in 100 patients admitted with pandemic influenza in four intensive care units in Uruguay during the winter of 2009. International Journal of Infectious Diseases. 2010;14:e85. PubMed PMID: 70125256.

47. Bagshaw SM, Sood MM, Long J, Fowler RA, Adhikari NK. Acute kidney injury among critically ill patients with pandemic H1N1 influenza A in Canada: cohort study. BMC Nephrol. 2013;14:123. Epub 2013/06/15. doi: 10.1186/1471-2369-14-123. PubMed PMID: 23763900; PubMed Central PMCID: PMC3694036.

48. Bahloul M, Dammak H, Chaari A, Allala R, Abid L, Haddar S, et al. Pulmonary capillary leak syndrome after influenza A (H1N1) virus infection. American Journal of Emergency Medicine. 2010;28(9):1063.e1-.e6. PubMed PMID: 2010605079.

49. Bai L, Gu L, Cao B, Zhai XL, Lu M, Lu Y, et al. Clinical features of pneumonia caused by 2009 influenza A(H1N1) virus in Beijing, China. Chest. 2011;139(5):1156-64. PubMed PMID: 2011254950.

50. Baird JS, Buet A, Hymes SR, Ravindranath TM, Zackai S, Cannon JM, et al. Comparing the clinical severity of the first versus second wave of 2009 Influenza A (H1N1) in a New York City pediatric healthcare facility. Pediatric Critical Care Medicine. 2012;13(4):375-80. PubMed PMID: 2012437310.

51. Bantar C, Oliva ME, Re HA, Sandillu M, Franco D, Izaguirre M, et al. Severe acute respiratory disease in the setting of an epidemic of swine-origin type A H1N1 influenza at a reference hospital in Entre Rios, Argentina. Clinical Infectious Diseases. 2009;49(9):1458-60. PubMed PMID: 19824855.

52. Bassetti M, Parisini A, Calzi A, Pallavicini FMB, Cassola G, Artioli S, et al. Risk factors for severe complications of the novel influenza A (H1N1): analysis of patients hospitalized in Italy. Clinical Microbiology & Infection. 2011;17(2):247-50. PubMed PMID: 20518797.

53. Bedford JR, Ivermee CL, Lange K, Chapman MJ. Nutrition and functional outcomes after critical illness due to pandemic (H1N1) 2009 influenza: a retrospective cohort study. Critical Care & Resuscitation. 2011;13(4):226-31. PubMed PMID: 22129283.

54. Belenguer-Muncharaz A, Reig-Valero R, Altaba-Tena S, Casero-Roig P, Ferrandiz-Selles A. Noninvasive mechanical ventilation in severe pneumonia due to H1N1 virus. [Spanish]

Utilizacion de la ventilacion mec\nica no invasiva en neumonia grave por virus H1N1. Medicina Intensiva. 2011;35(8):470-7. PubMed PMID: 2011554745.

55. Bertolini G, Rossi C, Crespi D, Finazzi S, Morandotti M, Rossi S, et al. Is influenza A(H1N1) pneumonia more severe than other community-acquired pneumonias? Results of the GiViTI survey of 155 Italian ICUs. Intensive Care Med. 2011;37(11):1746-55. PubMed PMID: 21847646.

56. Bessereau J, Chenaitia H, Michelet P, Roch A, Gariboldi V. Acute respiratory distress syndrome following 2009 H1N1 virus pandemic: when ECMO come to the patient bedside. Annales Francaises d Anesthesie et de Reanimation. 2010;29(2):165-6. PubMed PMID: 20153600.

57. Beurtheret S, Mastroianni C, Pozzi M, D'Alessandro C, Luyt C-E, Combes A, et al. Extracorporeal membrane oxygenation for 2009 influenza A (H1N1) acute respiratory distress syndrome: single-centre experience with 1-year follow-up. European Journal of Cardio-Thoracic Surgery. 2012;41(3):691-5. PubMed PMID: 22228837.

58. Blakemore SP, Gopalan PD. Pandemic influenza A (H1N1) 2009: A case series from intensive care units in port Shepstone, South Africa. Southern African Journal of Anaesthesia and Analgesia. 2010;16(3):17-22. PubMed PMID: 2010571959.

59. Blumental S, Huisman E, Cornet M-C, Ferreiro C, De Schutter I, Reynders M, et al. Pandemic A/H1N1v influenza 2009 in hospitalized children: a multicenter Belgian survey. BMC Infectious Diseases. 2011;11:313. PubMed PMID: 22060843; PubMed Central PMCID: PMCPMC3224785.

60. Bonastre J, Suberviola B, Pozo JC, Guerrero JE, Torres A, Rodriguez A, et al. [Extracorporeal lung support in patients with severe respiratory failure secondary to the 2010-2011 winter seasonal outbreak of influenza A (H1N1) in Spain]. Medicina Intensiva. 2012;36(3):193-9. PubMed PMID: 22341559.

61. Boots RJ, Lipman J, Lassig-Smith M, Stephens DP, Thomas J, Shehabi Y, et al. Experience with high frequency oscillation ventilation during the 2009 H1N1 influenza pandemic in Australia and New Zealand. Anaesthesia & Intensive Care. 2011;39(5):837-46. PubMed PMID: 21970127.

62. Borgatta B, Perez M, Vidaur L, Lorente L, Socias L, Pozo JC, et al. Elevation of creatine kinase is associated with worse outcomes in 2009 pH1N1 influenza A infection. Intensive Care Med. 2012;38(7):1152-61. PubMed PMID: 2012358163.

63. Bramley AM, Dasgupta S, Skarbinski J, Kamimoto L, Fry AM, Finelli L, et al. Intensive care unit patients with 2009 pandemic influenza A (H1N1pdm09) virus infection - United States, 2009. Influenza & Other Respiratory Viruses. 2012;6(6):e134-42. PubMed PMID: 22672249.

64. Brandsaeter BJ, Pillgram M, Berild D, Kjekshus H, Kran AMB, Bergersen BM. Hospitalised patients with suspected 2009 H1N1 Influenza A in a hospital in Norway, July - December 2009. BMC Infectious Diseases. 2011;11(75). PubMed PMID: 2011211810.

65. Brink M, Hagberg L, Larsson A, Gedeborg R. Respiratory support during the influenza A (H1N1) pandemic flu in Sweden. Acta Anaesthesiologica Scandinavica. 2012;56(8):976-86. Epub 2012/06/26. doi: 10.1111/j.1399-6576.2012.02727.x. PubMed PMID: 22724889.

66. Brown SM, Pittman J, Miller IRR, Horton KD, Markewitz B, Hirshberg E, et al. Right and left heart failure in severe H1N1 influenza A infection. European Respiratory Journal. 2011;37(1):112-8. PubMed PMID: 2011062949.

67. Brun-Buisson C, Richard JCM, Mercat A, Thiebaut ACM, Brochard L. Early corticosteroids in severe influenza A/H1N1 pneumonia and acute respiratory distress syndrome. Am J Respir Crit Care Med. 2011;183(9):1200-6. PubMed PMID: 2011238877.

68. Caler CL, Balsera EC, Lopez JLG, Saez RL. Severe acute respiratory failure in patients with influenza A (H1N1) virus infection admitted in intensive care. European Journal of Internal Medicine. 2010;21(5):469. PubMed PMID: 20816610.

69. Camous L, Lemiale V, Canet E, Max A, Schnell D, Le Goff J, et al. Clinical features of H1N1 2009 infection in critically ill immunocompromised patients. Critical Care (London, England). 2010;14(2):139. PubMed PMID: 20392286; PubMed Central PMCID: PMCPMC2887153.

70. Campbell A, Rodin R, Kropp R, Mao Y, Hong Z, Vachon J, et al. Risk of severe outcomes among patients admitted to hospital with pandemic (H1N1) influenza. CMAJ Canadian Medical Association Journal. 2010;182(4):349-55. PubMed PMID: 20159893; PubMed Central PMCID: PMCPMC2831689.

71. Canak G, Kovacevic N, Vukadinov J, Turkulov V, Sevic S, Doder R, et al. Clinical features, treatments and outcomes of influenza A (H1N1) 2009 among the hospitalized patients in the Clinic for Infectious Diseases in Novi Sad

Klinicke karakteristike, terapije i ishodi lecenja gripa A (H1N1) 2009. kod bolesnika koji su lezali u Klinici za infektivne bolesti u Novom Sadu. Vojnosanitetski Pregled. 2013;70(2):155-62. PubMed PMID: 2013089547.

72. Caprotta G, Gonzalez Crotti P, Primucci Y, Alesio H, Esen A. Influenza A H1N1 respiratory infection in an intensive care unit in Argentina. [Spanish]

Infeccion respiratoria por influenza A H1N1 en cuidados intensivos de la Republica Argentina. Anales de Pediatria. 2010;72(1):62-6. PubMed PMID: 2010024720.

73. Celjuska-Tosev E, Kuzman I, Drazenovic V, Knezovic I, Civljak R. Clinical and epidemiological characteristics of hospitalized patients with pandemic a(H1N1) 2009 influenza. [Croatian]

Klinicke i epidemioloske znacajke hospitaliziranih bolesnika s pandemijskom influencom a(H1N1) 2009. Infektoloski Glasnik. 2010;30(4):149-59. PubMed PMID: 2011246057.

74. Chacko B, Peter JV, Pichamuthu K, Ramakrishna K, Moorthy M, Karthik R, et al. Cardiac manifestations in patients with pandemic (H1N1) 2009 virus infection needing intensive care. Journal of Critical Care. 2012;27(1):106.e1-6. PubMed PMID: 21737242.

75. Chacko J, Gagan B, Ashok E, Radha M, Hemanth HV. Critically ill patients with 2009 H1N1 infection in an Indian ICU. Indian Journal of Critical Care Medicine. 2010;14(2):77-82. PubMed PMID: 2010465327.

76. Champunot R, Tanjatham S, Kerdsin A, Puangpatra P, Wangsai S, Treebuphachatsakul P, et al. Impact of pandemic influenza (H1N1) virus-associated community-acquired pneumonia among adults in a tertiary hospital in Thailand. Jpn J Infect Dis. 2010;63(4):251-6. Epub 2010/07/27. PubMed PMID: 20657064.

77. Jaber S, Conseil M, Coisel Y, Jung B, Chanques G. [ARDS and influenza A (H1N1): patients' characteristics and management in intensive care unit. A literature review]. Ann Fr Anesth Reanim. 2010;29(2):117-25. Epub 2010/02/02. doi: 10.1016/j.annfar.2009.12.026. PubMed PMID: 20116970.

78. Chang YS, Van Hal SJ, Spencer PM, Gosbell IB, Collett PW. Comparison of adult patients hospitalised with pandemic (H1N1) 2009 influenza and seasonal influenza during the "PROTECT" phase of the pandemic response. Medical Journal of Australia. 2010;192(2):90-3. PubMed PMID: 2010281020.

79. Chien JMF, Tan BH, Yang KS, Tan TT, Low CY, Kurup A, et al. Severe infection with H1N1 requiring intensive care - lessons for preparedness programmes. Annals of the Academy of Medicine Singapore. 2010;39(4):328-32. PubMed PMID: 2010283058.

80. Chien Y-S, Su C-P, Tsai H-T, Huang AS, Lien C-E, Hung M-N, et al. Predictors and outcomes of respiratory failure among hospitalized pneumonia patients with 2009 H1N1 influenza in Taiwan. Journal of Infection. 2010;60(2):168-74. PubMed PMID: 20036689.

81. Choi EY, Huh JW, Lim CM, Koh Y, Kim SH, Choi SH, et al. Critically ill patients with pandemic influenza A/H1N1 2009 at a Medical Center in Korea. Tuberculosis and Respiratory Diseases. 2011;70(1):28-35. PubMed PMID: 2011123483.

82. Cianchi G, Bonizzoli M, Pasquini A, Bonacchi M, Zagli G, Ciapetti M, et al. Ventilatory and ECMO treatment of H1N1-induced severe respiratory failure: results of an Italian referral ECMO center. BMC Pulmonary Medicine. 2011;11:2. PubMed PMID: 21223541; PubMed Central PMCID: PMCPMC3022902.

83. Cilloniz C, Ewig S, Menendez R, Ferrer M, Polverino E, Reyes S, et al. Bacterial co-infection with H1N1 infection in patients admitted with community acquired pneumonia. Journal of Infection. 2012;65(3):223-30. PubMed PMID: 22543245.

84. Cordero E, Perez-Romero P, Moreno A, Len O, Montejo M, Vidal E, et al. Pandemic influenza A(H1N1) virus infection in solid organ transplant recipients: Impact of viral and non-viral co-infection. Clinical Microbiology and Infection. 2012;18(1):67-73. PubMed PMID: 2011694695.

85. Cornejo R, Tobar E, Diaz G, Romero C, Llanos O, Galvez LR, et al. Systematic approach for severe respiratory failure due to novel A (H1N1) influenza. Minerva Anestesiologica. 2011;77(5):510-21. PubMed PMID: 21540806.

86. Creanga AA, Kamimoto L, Newsome K, D'Mello T, Jamieson DJ, Zotti ME, et al. Seasonal and 2009 pandemic influenza A (H1N1) virus infection during pregnancy: a population-based study of hospitalized cases. Am J Obstet Gynecol. 2011;204(6 Suppl 1):S38-45. Epub 2011/04/22. doi: 10.1016/j.ajog.2011.02.037. PubMed PMID: 21507375.

87. Cullen G, Martin J, O'Donnell J, Boland M, Canny M, Keane E, et al. Surveillance of the first 205 confirmed hospitalised cases of pandemic H1N1 influenza in Ireland, 28 April - 3 October 2009.[Erratum appears in Euro Surveill. 2009;14(45) pii: 19398]. Euro Surveillance: Bulletin Europeen sur les Maladies Transmissibles = European Communicable Disease Bulletin. 2009;14(44). PubMed PMID: 19941779.

88. Cuquemelle E, Soulis F, Villers D, Roche-Campo F, Ara Somohano C, Fartoukh M, et al. Can procalcitonin help identify associated bacterial infection in patients with severe influenza pneumonia? A multicentre study. Intensive Care Med. 2011;37(5):796-800. PubMed PMID: 21369807.

89. Curcio D, Ferreira Cabrera L, Duarte A, Valencia E, Paz Chavez CH, Ibanez-Guzman C, et al. Ventilator-associated pneumonia in patients with 2009 pandemic influenza A (H1N1) infection: an observational study. J Chemother. 2010;22(6):428-30. Epub 2011/02/10. doi: 10.1179/joc.2010.22.6.428. PubMed PMID: 21303753.

90. Custodio HT, Gayle MO, Bailey CS, Wludyka PS, Rathore MH. Comparison of ICU and non-ICU patients infected with the 2009 H1N1 influenza virus in a Florida Children's hospital between April and December 2009. Eastern Journal of Medicine. 2011;16(3):188-93. PubMed PMID: 2011572621.

91. D'Ancona G, Capitanio G, Chiaramonte G, Serretta R, Turrisi M, Pilato M, et al. Extracorporeal membrane oxygenator rescue and airborne transportation of patients with influenza A (H1N1) acute respiratory distress syndrome in a Mediterranean underserved area. Interactive Cardiovascular & Thoracic Surgery. 2011;12(6):935-7. PubMed PMID: 21441254.

92. Damak H, Chtara K, Bahloul M, Kallel H, Ksibi H, Chaari A, et al. Clinical features, complications and mortality in critically ill patients with 2009 influenza A(H1N1) in Sfax,Tunisia. Influenza Other Respir Viruses. 2011;5(4):230-40. PubMed PMID: 2011323266.

93. del Rosal T, Baquero-Artigao F, Calvo C, Mellado MJ, Molina JC, Santos MdM, et al. Pandemic H1N1 influenza-associated hospitalizations in children in Madrid, Spain. Influenza & Other Respiratory Viruses. 2011;5(6):e544-51. PubMed PMID: 21781285.

94. Deng LH, Zeng YL, Feng P, Liu YL, Wang LC, Bai Y, et al. Clinical characteristics of critical patients with pandemic influenza a (H1N1) virus infection in Chengdu, China. Journal of Zhejiang University: Science B. 2012;13(1):49-55. PubMed PMID: 2012084611.

95. Diaz E, Rodriguez A, Martin-Loeches I, Lorente L, del Mar Martin M, Pozo JC, et al. Impact of obesity in patients infected with 2009 influenza A(H1N1). Chest. 2011;139(2):382-6. PubMed PMID: 20688928.

96. Diaz E, Martin-Loeches I, Canadell L, Vidaur L, Suarez D, Socias L, et al. Corticosteroid therapy in patients with primary viralpneumonia due to pandemic (H1N1) 2009influenza. Journal of Infection. 2012;64(3):311-8. PubMed PMID: 2012075615.

97. Djordjevic Z, Lazic Z, Gajovic O, Canovic P, Todorovic Z, Mijailovic Z, et al. [Risk factors for acute respiratory distress syndrome development in patients with type A influenza (H1N1)]. Srpski Arhiv Za Celokupno Lekarstvo. 2012;140(7-8):441-7. PubMed PMID: 23092028.

98. Dominguez-Cherit G, Lapinsky SE, Macias AE, Pinto R, Espinosa-Perez L, de la Torre A, et al. Critically Ill patients with 2009 influenza A(H1N1) in Mexico. JAMA. 2009;302(17):1880-7. PubMed PMID: 19822626.

99. Duarte PA, Venazzi A, Youssef NC, Oliveira MC, Tannous LA, Duarte CB, et al. Outcome of influenza A (H1N1) patients admitted to intensive care units in the Parana state, Brazil. Rev Bras Ter Intensiva. 2009;21(3):231-6. Epub 2009/08/01. PubMed PMID: 25303543.

100. Dubar G, Azria E, Tesniere A, Dupont H, Le Ray C, Baugnon T, et al. French experience of 2009 A/H1N1v influenza in pregnant women. PLoS One. 2010;5(10). PubMed PMID: 2010598928.

101. Efstathiou. Deaths and hospitalizations related to 2009 pandemic influenza A (H1N1) - Greece, May 2009-February 2010. MMWR Morb Mortal Wkly Rep. 2010;59(22):682-6. Epub 2010/06/11. PubMed PMID: 20535092.

102. Ellington SR, Hartman LK, Acosta M, Martinez-Romo M, Rubinson L, Jamieson DJ, et al. Pandemic 2009 influenza A (H1N1) in 71 critically ill pregnant women in California. American Journal of Obstetrics & Gynecology. 2011;204(6 Suppl 1):S21-30. PubMed PMID: 21514554.

103. Estenssoro E, Rios FG, Apezteguia C, Reina R, Neira J, Ceraso DH, et al. Pandemic 2009 influenza A in Argentina: a study of 337 patients on mechanical ventilation. Am J Respir Crit Care Med. 2010;182(1):41-8. Epub 2010/03/06. doi: 10.1164/201001-0037OC. PubMed PMID: 20203241.

104. Fagnoul D, Pasquier P, Bodson L, Ortiz JA, Vincent JL, De Backer D. Cardiovascular dysfunction in severe influenza (H1N1) infection. Intensive Care Med. 2011;37:S154. PubMed PMID: 70639404.

105. Farias JA, Fernandez A, Monteverde E, Vidal N, Arias P, Montes MJ, et al. Critically ill infants and children with influenza A (H1N1) in pediatric intensive care units in Argentina. Intensive Care Med. 2010;36(6):1015-22. PubMed PMID: 20237757.

106. Freed DH, Henzler D, White CW, Fowler R, Zarychanski R, Hutchison J, et al. Extracorporeal lung support for patients who had severe respiratory failure secondary to influenza A (H1N1) 2009 infection in Canada. Canadian Journal of Anesthesia. 2010;57(3):240-7. PubMed PMID: 2010321338.

107. Fuhrman C, Bonmarin I, Bitar D, Cardoso T, Duport N, Herida M, et al. Adult intensive-care patients with 2009 pandemic influenza A(H1N1) infection. Epidemiol Infect. 2011;139(8):1202-9. PubMed PMID: 20974021.

108. Garnacho-Montero J, Gutierrez-Pizarraya A, Marquez JA, Zaragoza R, Granada R, Ruiz-Santana S, et al. Epidemiology, clinical features, and prognosis of elderly adults with severe forms of influenza A (H1N1. Journal of the American Geriatrics Society. 2013;61(3):350-6. PubMed PMID: 23496351.

109. Gauzere BA, Malvy D, Filleul L, Ramful D, Jaffar-Bandjee MC, El Bock M, et al. Intensive care unit admission for pandemic (H1N1) 2009, Reunion Island, 2009. Emerg Infect Dis. 2011;17(1):140-1. PubMed PMID: 2011019155.

110. Gilca R, De Serres G, Boulianne N, Ouhoummane N, Papenburg J, Douville-Fradet M, et al. Risk factors for hospitalization and severe outcomes of 2009 pandemic H1N1 influenza in Quebec, Canada. Influenza & Other Respiratory Viruses. 2011;5(4):247-55. PubMed PMID: 21651735.

111. Gomez-Gomez A, Magana-Aquino M, Garcia-Sepulveda CA, Ochoa-Perez UR, Falcon-Escobedo R, Comas-Garcia A, et al. Severe pneumonia associated with pandemic (H1N1) 2009 outbreak, San Luis Potosi, Mexico. Emerg Infect Dis. 2010;16(1):27-34. PubMed PMID: 2010035596.

112. Gonzalez-Velez AE, Diaz-Agero-Perez C, Robustillo-Rodela A, Cornejo-Gutierrez AM, Pita-Lopez MJ, Oliva-Iniguez L, et al. Factors associated to admission to Intensive Care in patients hospitalized due to pandemic Influenza A/H1N1 2009. [Spanish]

Factores asociados a ingreso en unidad de cuidados intensivos en pacientes hospitalizados por Influenza pandemica A/H1N1 2009. Medicina Intensiva. 2011;35(8):463-9. PubMed PMID: 2011554744.

113. Gordon CL, Langan K, Charles PGP, Bellomo R, Hart GK, Torresi J, et al. Pooled human immunoglobulin therapy in critically Ill patients with pandemic 2009 influenza A(H1N1) pneumonitis and immunoglobulin G2 subclass (IgG2) deficiency. Clinical Infectious Diseases. 2011;52(3):422-6. PubMed PMID: 21217191.

114. Grasselli G, Bombino M, Patroniti N, Foti G, Benini A, Abbruzzese C, et al. Management of acute respiratory complications from influenza A (H1N1) infection: experience of a tertiarylevel Intensive Care Unit. Minerva Anestesiologica. 2011;77(9):884-91. PubMed PMID: 21878870.

115. Grasso S, Terragni P, Birocco A, Urbino R, Del Sorbo L, Filippini C, et al. ECMO criteria for influenza A (H1N1)-associated ARDS: role of transpulmonary pressure. Intensive Care Med. 2012;38(3):395-403. PubMed PMID: 22323077.

116. Gubbels S, Perner A, Valentiner-Branth P, Molbak K. National surveillance of pandemic influenza A(H1N1) infection-related admissions to intensive care units during the 2009-10 winter peak in Denmark: two complementary approaches. Euro Surveillance: Bulletin Europeen sur les Maladies Transmissibles = European Communicable Disease Bulletin. 2010;15(49):9. PubMed PMID: 21163180.

117. Hagau N, Slavcovici A, Gonganau DN, Oltean S, Dirzu DS, Brezoszki ES, et al. Clinical aspects and cytokine response in severe H1N1 influenza A virus infection. Crit Care. 2009;13(6). PubMed PMID: 2010709459.

118. Hajjar LA, Mauad T, Galas FRBG, Kumar A, da Silva LFF, Dolhnikoff M, et al. Severe novel influenza A (H1N1) infection in cancer patients. Annals of Oncology. 2010;21(12):2333-41. PubMed PMID: 20511340; PubMed Central PMCID: PMCPMC2990816.

119. Hammond NE, Corley A, Fraser JF. The utility of procalcitonin in diagnosis of H1N1 influenza in intensive care patients. Anaesthesia & Intensive Care. 2011;39(2):238-41. PubMed PMID: 21485672.

120. Han K, Ma H, An X, Su Y, Chen J, Lian Z, et al. Early use of glucocorticoids was a risk factor for critical disease and death from pH1N1 infection. Clinical Infectious Diseases. 2011;53(4):326-33. PubMed PMID: 2011436553.

121. Hernandez JE, Adiga R, Armstrong R, Bazan J, Bonilla H, Bradley J, et al. Clinical experience in adults and children treated with intravenous peramivir for 2009 influenza A (H1N1) under an emergency IND program in the United States. Clinical Infectious Diseases. 2011;52(6):695-706. PubMed PMID: 2011190871.

122. Hernandez-Bou S, Novell CB, Alins JG, Garcia-Garcia JJ, Infectious Diseases Working Group SSoPE. Hospitalized children with influenza A H1N1 (2009) infection: a Spanish multicenter study. Pediatric Emergency Care. 2013;29(1):49-52. PubMed PMID: 23283263.

123. Holzgraefe B, Broome M, Kalzen H, Konrad D, Palmer K, Frenckner B. Extracorporeal membrane oxygenation for pandemic H1N1 2009 respiratory failure. Minerva Anestesiologica. 2010;76(12):1043-51. PubMed PMID: 21178913.

124. Hong SB, Choi EY, Kim SH, Suh GY, Park MS, Lee MG, et al. Epidemiological analysis of critically ill adult patients with pandemic influenza A(H1N1) in South Korea. Epidemiology & Infection. 2013;141(5):1070-9. PubMed PMID: 22853817.

125. Hou X, Guo L, Zhan Q, Jia X, Mi Y, Li B, et al. Extracorporeal membrane oxygenation for critically ill patients with 2009 influenza A (H1N1)-related acute respiratory distress syndrome: preliminary experience from a single center. Artificial Organs. 2012;36(9):780-6. PubMed PMID: 22747918.

126. Ismail HIM, Tan KK, Lee YL, Pau WSC, Razali KAM, Mohamed T, et al. Characteristics Of children hospitalized for pandemic (H1N1) 2009, Malaysia. Emerg Infect Dis. 2011;17(4):708-10. PubMed PMID: 2011189579.

127. Jain S, Kamimoto L, Bramley AM, Schmitz AM, Benoit SR, Louie J, et al. Hospitalized patients with 2009 H1N1 influenza in the United States, April-June 2009. New England Journal of Medicine. 2009;361(20):1935-44. PubMed PMID: 19815859.

128. Jog S, Patel D, Dravid T, Rajhans P, Akole P, Pawar B, et al. Early application of high frequency oscillatory ventilation in 'H1N1 influenza' related ARDS is associated with better outcome: A retrospective study. Intensive Care Med. 2013;39(6):1146-7. PubMed PMID: 2013320231.

129. Jouvet P, Hutchison J, Pinto R, Menon K, Rodin R, Choong K, et al. Critical illness in children with influenza A/pH1N1 2009 infection in Canada. Pediatric Critical Care Medicine. 2010;11(5):603-9. PubMed PMID: 2010552644.

130. Jung JY, Park BH, Hong SB, Koh Y, Suh GY, Jeon K, et al. Acute kidney injury in critically ill patients with pandemic influenza A pneumonia 2009 in Korea: A multicenter study. Journal of Critical Care. 2011;26(6):577-85. PubMed PMID: 2011648076.

131. Kendirli T, Demirkol D, Yldzdas D, Anl AB, Asloglu N, Karapnar B, et al. Critically ill children with pandemic influenza (H1N1) in pediatric intensive care units in Turkey. Pediatric Critical Care Medicine. 2012;13(1):e11-e7. PubMed PMID: 2012026743.

132. Kim SH, Hong SB, Yun SC, Choi WI, Ahn JJ, Lee YJ, et al. Corticosteroid treatment in critically ill patients with pandemic influenza A/H1N1 2009 infection: Analytic strategy using propensity scores. Am J Respir Crit Care Med. 2011;183(9):1207-14. PubMed PMID: 2011238878.

133. Kim W-Y, Young Suh G, Huh JW, Kim S-H, Kim M-j, Kim YS, et al. Triple-combination antiviral drug for pandemic H1N1 influenza virus infection in critically ill patients on mechanical ventilation. Antimicrobial Agents & Chemotherapy. 2011;55(12):5703-9. PubMed PMID: 21968371; PubMed Central PMCID: PMCPMC3232815.

134. Knight M, Pierce M, Seppelt I, Kurinczuk JJ, Spark P, Brocklehurst P, et al. Critical illness with AH1N1v influenza in pregnancy: a comparison of two population-based cohorts. BJOG: An International Journal of Obstetrics & Gynaecology. 2011;118(2):232-9. PubMed PMID: 21040393.

135. Kobayashi M, Ohfuji S, Fukushima W, Sugiura S, Kohdera U, Itoh Y, et al. Pediatric hospitalizations with influenza A infection during the 2009-2010 pandemic in five hospitals in Japan. Pediatrics International. 2012;54(5):613-8. PubMed PMID: 2012581077.

136. Koegelenberg CFN, Irusen EM, Cooper R, Diacon AH, Taljaard JJ, Mowlana A, et al. High mortality from respiratory failure secondary to swine-origin influenza A (H1N1) in South Africa. Qjm. 2010;103(5):319-25. PubMed PMID: 20219780.

137. Kojicic M, Kovacevic P, Bajramovic N, Batranovic U, Vidovic J, Aganovic K, et al. Characteristics and outcome of mechanically ventilated patients with 2009 H1N1 influenza in Bosnia and Herzegovina and Serbia: Impact of newly established multidisciplinary intensive care unit. Croatian Medical Journal. 2012;53(6):620-6. PubMed PMID: 2013060856.

138. Kopel E, Amitai Z, Grotto I, Kaliner E, Volovik I. Patients with pandemic (H1N1) 2009 in intensive care units, Israel. Emerg Infect Dis. 2010;16(4):720-1. PubMed PMID: 20350400; PubMed Central PMCID: PMCPMC3321970.

139. Kumar A, Zarychanski R, Pinto R, Cook DJ, Marshall J, Lacroix J, et al. Critically ill patients with 2009 influenza A(H1N1) infection in Canada. JAMA. 2009;302(17):1872-9. PubMed PMID: 19822627.

140. Kumar TCN, Shivakumar NS, Deepak TS, Rashmi K, Goutam MS, Vivek G. H1N1-infected patients in ICU and heir clinical outcome. North American Journal of Medical Sciences. 2012;4(9):394-8. PubMed PMID: 2012556269.

141. Kute VB, Godara SM, Goplani KR, Gumber MR, Shah PR, Vanikar AV, et al. High mortality in critically ill patients infected with 2009 pandemic influenza A (H1N1) with pneumonia and acute kidney injury. Saudi Journal of Kidney Diseases & Transplantation. 2011;22(1):83-9. PubMed PMID: 21196619.

142. Labeaud AD, Wentworth B, Gildengorin G, Tam K, Guardia-Labar L, Petru A. Comparison of moderate and severe hospitalized pediatric 2009 H1N1 influenza cases. Pediatric Infectious Disease Journal. 2013;32(2):e90-e3. PubMed PMID: 2013069053.

143. Lai AR, Keet K, Yong CM, Diaz JV. Severe H1N1-Associated acute respiratory distress syndrome: A case series. American Journal of Medicine. 2010;123(3):282-5.e2. PubMed PMID: 20193840.

144. Lam J, Nikhanj N, Ngab T, Tennant R, Shahedi K, Mathisen G, et al. Severe cases of pandemic H1N1 pneumonia and respiratory failure requiring intensive care. Journal of Intensive Care Medicine. 2011;26(5):318-25. PubMed PMID: 2011510118.

145. Lazoura O, Parthipun AA, Roberton BJ, Downey K, Finney S, Padley S. Acute respiratory distress syndrome related to influenza A H1N1 infection: correlation of pulmonary computed tomography findings to extracorporeal membrane oxygenation treatment and clinical outcome. Journal of Critical Care. 2012;27(6):602-8. PubMed PMID: 22699031.

146. Leen T, Williams TA, Campbell L, Chamberlain J, Gould A, McEntaggart G, et al. Early experience with influenza A H1N109 in an Australian intensive care unit. Intensive & Critical Care Nursing. 2010;26(4):207-14. PubMed PMID: 20599382.

147. Linko R, Pettila V, Ruokonen E, Varpula T, Karlsson S, Tenhunen J, et al. Corticosteroid therapy in intensive care unit patients with PCR-confirmed influenza A(H1N1) infection in Finland. Acta Anaesthesiologica Scandinavica. 2011;55(8):971-9. PubMed PMID: 2011485341.

148. Lister P, Reynolds F, Parslow R, Chan A, Cooper M, Plunkett A, et al. Swine-origin influenza virus H1N1, seasonal influenza virus, and critical illness in children. Lancet. 2009;374(9690):605-7. PubMed PMID: 19700000.

149. Liu L, Zhang R-F, Lu H-Z, Lu S-H, Huang Q, Xiong Y-Y, et al. Sixty-two severe and critical patients with 2009 influenza A (H1N1) in Shanghai, China. Chinese Medical Journal. 2011;124(11):1662-6. PubMed PMID: 21740773.

150. Liu J, Li Q, Cui H, Liu C. Severe and critical cases of H1N1 influenza in pregnancy: a Chinese perspective. Journal of Postgraduate Medicine. 2011;57(4):298-301. PubMed PMID: 22120858.

151. Lockman JL, Fischer WA, Perl TM, Valsamakis A, Nichols DG. The critically ill child with novel H1N1 influenza A: a case series. Pediatric Critical Care Medicine. 2010;11(2):173-8. PubMed PMID: 20048690.

152. Lopez-Delgado JC, Rovira A, Esteve F, Rico N, Mendiluce RM, Noguera JB, et al. Thrombocytopenia as a mortality risk factor in acute respiratory failure in H1N1 influenza. Swiss Medical Weekly. 2013;143(w13788). PubMed PMID: 2013304494.

153. Louie JK, Acosta M, Jamieson DJ, Honein MA, California Pandemic Working G. Severe 2009 H1N1 influenza in pregnant and postpartum women in California. New England Journal of Medicine. 2010;362(1):27-35. PubMed PMID: 20032319.

154. Louie JK, Yang S, Acosta M, Yen C, Samuel MC, Schechter R, et al. Treatment with neuraminidase inhibitors for critically ill patients with influenza A (H1N1)pdm09. Clinical Infectious Diseases. 2012;55(9):1198-204. PubMed PMID: 22843781.

155. Louriz M, Mahraoui C, Azzouzi A, El Fassy Fihri MT, Zeggwagh AA, Abidi K, et al. Clinical features of the initial cases of 2009 pandemic influenza A (H1N1) virus infection in an university hospital of Morocco. International Archives of Medicine. 2010;3(1). PubMed PMID: 2010645510.

156. Mady A, Ramadan OS, Yousef A, Mandourah Y, Amr AA, Kherallah M. Clinical experience with severe 2009 H1N1 influenza in the intensive care unit at King Saud Medical City, Saudi Arabia. Journal of Infection and Public Health. 2012;5(1):52-6. PubMed PMID: 22341843.

157. Maravi-Poma E, Martin-Loeches I, Regidor E, Laplaza C, Cambra K, Aldunate S, et al. Severe 2009 A/H1N1v influenza in pregnant women in Spain. Crit Care Med. 2011;39(5):945-51. PubMed PMID: 21263317.

158. Martin SS, Hollingsworth CL, Norfolk SG, Wolfe CR, Hollingsworth JW. Reversible cardiac dysfunction associated with pandemic 2009 influenza A(H1N1). Chest. 2010;137(5):1195-7. PubMed PMID: 2010260245.

159. Martin-Loeches I, Diaz E, Vidaur L, Torres A, Laborda C, Granada R, et al. Pandemic and post-pandemic Influenza A (H1N1) infection in critically ill patients. Critical Care. 2011;15(6). PubMed PMID: 2012084266.

160. Martin-Loeches I, Papiol E, Rodriguez A, Diaz E, Zaragoza R, Granada RM, et al. Acute kidney injury in critical ill patients affected by influenza A (H1N1) virus infection. Critical Care (London, England). 2011;15(1):R66. PubMed PMID: 21342489; PubMed Central PMCID: PMCPMC3221999.

161. Martin-Loeches I, Sanchez-Corral A, Diaz E, Granada RM, Zaragoza R, Villavicencio C, et al. Community-acquired respiratory coinfection in critically ill patients with pandemic 2009 influenza A(H1N1) virus. Chest. 2011;139(3):555-62. PubMed PMID: 20930007.

162. Masclans JR, Perez M, Almirall J, Lorente L, Marques A, Socias L, et al. Early non-invasive ventilation treatment for severe influenza pneumonia. Clinical Microbiology and Infection. 2013;19(3):249-56. PubMed PMID: 2013128707.

163. Miller IRR, Markewitz BA, Rolfs RT, Brown SM, Dascomb KK, Grissom CK, et al. Clinical findings and demographic factors associated with ICU admission in utah due to novel 2009 influenza a(H1N1) infection. Chest. 2010;137(4):752-8. PubMed PMID: 2010220639.

164. Morgan CI, Hobson MJ, Seger B, Rice MA, Staat MA, Wheeler DS. 2009 pandemic influenza A (H1N1) in critically ill children in Cincinnati, Ohio. Pediatric Critical Care Medicine. 2012;13(3):e140-4. PubMed PMID: 21760562.

165. Muscedere J, Ofner M, Kumar A, Long J, Lamontagne F, Cook D, et al. The occurrence and impact of bacterial organisms complicating critical care illness associated with 2009 influenza A(H1N1) infection. Chest. 2013;144(1):39-47. Epub 2013/02/09. doi: 10.1378/chest.12-1861. PubMed PMID: 23392627.

166. Nair P, Davies AR, Beca J, Bellomo R, Ellwood D, Forrest P, et al. Extracorporeal membrane oxygenation for severe ARDS in pregnant and postpartum women during the 2009 H1N1 pandemic. Intensive Care Med. 2011;37(4):648-54. PubMed PMID: 21318437.

167. Naseem A, Satti S, Khan MA, Saeed W. A clinical account of hospitalized 2009 pandemic influenza A (H1N1) cases. J Coll Physicians Surg Pak. 2011;21(2):97-102. Epub 2011/02/22. doi: 02.2011/JCPSP.97102. PubMed PMID: 21333241.

168. Ni Z, Guo Z, Chen X, Wang Q, Qiu Y, Wu T, et al. Cardiac injury in patients with pandemic 2009 influenza A (H1N1) infection. Acta Cardiologica. 2011;66(4):427-32. PubMed PMID: 21894797.

169. Nicolay N, Callaghan MA, Domegan LM, Oza AN, Marsh BJ, Flanagan PC, et al. Epidemiology, clinical characteristics and resource implications of pandemic (H1N1) 2009 in intensive care units in Ireland. Critical Care & Resuscitation. 2010;12(4):255-61. PubMed PMID: 21143086.

170. Nicolini A, Claudio S, Rao F, Ferrera L, Isetta M, Bonfiglio M. Influenza A (H1N1)-associated pneumonia

Pneumonia associada a influenza A (H1N1). Jornal Brasileiro de Pneumologia. 2011;37(5):621-7. PubMed PMID: 22042394.

171. Nin N, Lorente JA, Soto L, Rios F, Hurtado J, Arancibia F, et al. Acute kidney injury in critically ill patients with 2009 influenzaA (H1N1) viral pneumonia: an observational study. Intensive Care Med. 2011;37(5):768-74. PubMed PMID: 21394630.

172. Nin N, Soto L, Hurtado J, Lorente JA, Buroni M, Arancibia F, et al. Clinical characteristics and outcomes of patients with 2009 influenza A(H1N1) virus infection with respiratory failure requiring mechanical ventilation. Journal of Critical Care. 2011;26(2):186-92. Epub 2010/08/07. doi: 10.1016/j.jcrc.2010.05.031. PubMed PMID: 20688465.

173. Noah MA, Peek GJ, Finney SJ, Griffiths MJ, Harrison DA, Grieve R, et al. Referral to an extracorporeal membrane oxygenation center and mortality among patients with severe 2009 influenza A(H1N1). JAMA - Journal of the American Medical Association. 2011;306(15):1659-68. PubMed PMID: 2011580011.

174. Norfolk SG, Hollingsworth CL, Wolfe CR, Govert JA, Que LG, Cheifetz IM, et al. Rescue therapy in adult and pediatric patients with pH1N1 influenza infection: a tertiary center intensive care unit experience from April to October 2009. Crit Care Med. 2010;38(11):2103-7. PubMed PMID: 20711068.

175. O Brien FJ, Jairam SD, Traynor CA, Kennedy CM, Power M, Denton MD, et al. Pandemic H1N1 (2009) and renal failure: the experience of the Irish national tertiary referral centre. Irish Journal of Medical Science. 2011;180(1):135-8. PubMed PMID: 20960237.

176. Oh DK, Lee MG, Choi EY, Lim J, Lee HK, Kim SC, et al. Low-tidal volume mechanical ventilation in patients with acute respiratory distress syndrome caused by pandemic influenza A/H1N1 infection. Journal of Critical Care. 2013;28(4):358-64. Epub 2013/04/23. doi: 10.1016/j.jcrc.2013.03.001. PubMed PMID: 23602273.

177. Okumura A, Nakagawa S, Kawashima H, Morichi S, Muguruma T, Saito O, et al. Severe form of encephalopathy associated with 2009 pandemic influenza A (H1N1) in Japan. Journal of Clinical Virology. 2013;56(1):25-30. PubMed PMID: 2012750805.

178. Olafson K, Ramsey CD, Ariano RE, Stasiuk A, Siddiqui F, Wong D, et al. Sedation and analgesia usage in severe pandemic H1N1 (2009) infection: A comparison to respiratory failure secondary to other infectious pneumonias

Uso de Sedantes y Analgesicos en la Infeccion Severa Pandemica H1N1 (2009): Comparacion con Fallo Respiratorio Secundario a Otras Pulmonias Infecciosas. Annals of Pharmacotherapy. 2012;46(1):9-20. PubMed PMID: 2012027277.

179. Pabst D, Kuehn J, Schuler-Luettmann S, Wiebe K, Lebiedz P. Acute Respiratory Distress Syndrome as a presenting manifestation in young patients infected with H1N1 influenza virus. European Journal of Internal Medicine. 2011;22(6):e119-24. PubMed PMID: 22075296.

180. Paiva MBS, Botoni FA, Teixeira Jr AL, de Miranda AS, de Oliveira CRA, Abrahao JO, et al. The behavior and diagnostic utility of procalcitonin and five other inflammatory molecules in critically ill patients with respiratory distress and suspected 2009 influenza a H1N1 infection. Clinics. 2012;67(4):327-34. PubMed PMID: 22522757.

181. Paredes G, Cevallos C. Acute respiratory distress syndrome during the 2009 H1N1 influenza A pandemic in Ecuador. [Spanish]

Sindrome de Distres Respiratorio Agudo, durante la pandemia de Influenza A H1N1 2009 en Ecuador. Medicina Intensiva. 2010;34(5):310-7. PubMed PMID: 2010339280.

182. Patroniti N, Zangrillo A, Pappalardo F, Peris A, Cianchi G, Braschi A, et al. The Italian ECMO network experience during the 2009 influenza A(H1N1) pandemic: preparation for severe respiratory emergency outbreaks. Intensive Care Med. 2011;37(9):1447-57. PubMed PMID: 21732167.

183. Pereira JM, Moreno RP, Matos R, Rhodes A, Martin-Loeches I, Cecconi M, et al. Severity assessment tools in ICU patients with 2009 influenza A (H1N1) pneumonia. Clinical Microbiology & Infection. 2012;18(10):1040-8. PubMed PMID: 22264290.

184. Perez Navero JL, Rumbao Aguirre J, Correas Sanchez A, Saldana Garcia N, Munoz-Villanueva MC, Ibarra De La Rosa I. Clinical characteristics of patients with infection due to Influenza A (H1N1) 2009 and critical pathology. [Spanish]

Caracteristicas clinicas de pacientes con infeccion por Influenza A (H1N1) 2009 y patologia critica. Anales de Pediatria. 2011;74(2):97-102. PubMed PMID: 2011119116.

185. Perez-Padilla R, de la Rosa-Zamboni D, Ponce de Leon S, Hernandez M, Quinones-Falconi F, Bautista E, et al. Pneumonia and respiratory failure from swine-origin influenza A (H1N1) in Mexico. N Engl J Med. 2009;361(7):680-9. PubMed PMID: 19564631.

186. Petersen E, Keld DB, Ellermann-Eriksen S, Gubbels S, Ilkjaer S, Jensen-Fangel S, et al. Failure of combination oral oseltamivir and inhaled zanamivir antiviral treatment in ventilator- and ECMO-treated critically ill patients with pandemic influenza A (H1N1)v. Scandinavian Journal of Infectious Diseases. 2011;43(6-7):495-503. PubMed PMID: 21309638.

187. Pham T, Combes A, Roze H, Chevret S, Mercat A, Roch A, et al. Extracorporeal membrane oxygenation for pandemic influenza A(H1N1)-induced acute respiratory distress syndrome: a cohort study and propensity-matched analysis. Am J Respir Crit Care Med. 2013;187(3):276-85. Epub 2012/11/17. doi: 10.1164/rccm.201205-0815OC. PubMed PMID: 23155145.

188. Piacentini E, Sanchez B, Arauzo V, Calbo E, Cuchi E, Nava JM. Procalcitonin levels are lower in intensive care unit patients with H1N1 influenza A virus pneumonia than in those with community-acquired bacterial pneumonia. A pilot study. Journal of Critical Care. 2011;26(2):201-5. PubMed PMID: 20813489.

189. Power CA, Van Heerden PV, Moxon D, Martindale G, Roberts B. Extracorporeal membrane oxygenation for critically ill patients with influenza A (H1N1) 2009: a case series. Critical care and resuscitation : journal of the Australasian Academy of Critical Care Medicine. 2011;13(1):38-43. PubMed PMID: 21355828.

190. Quispe-Laime AM, Bracco JD, Barberio PA, Campagne CG, Rolfo VE, Umberger R, et al. H1N1 influenza A virus-associated acute lung injury: response to combination oseltamivir and prolonged corticosteroid treatment. Intensive Care Med. 2010;36(1):33-41. PubMed PMID: 19924393.

191. Ramakrishna K, Peter JV, Karthik G, Abraham AM, Surekha V, Karthik R, et al. InfluenzaA (H1N1) 2009 pandemic: was there a difference in the two waves in patients requiring admission to the intensive-care unit? Clinical Microbiology & Infection. 2011;17(9):1355-8. PubMed PMID: 21679373.

192. Ramakrishna K, Sampath S, Chacko J, Chacko B, Narahari DL, Veerendra HH, et al. Clinical profile and predictors of mortality of severe pandemic (H1N1) 2009 virus infection needing intensive care: A multi-centre prospective study from South India. Journal of Global Infectious Diseases. 2012;4(3):145-52. PubMed PMID: 2012551379.

193. Randolph AG, Vaughn F, Sullivan R, Rubinson L, Thompson BT, Yoon G, et al. Critically ill children during the 2009-2010 influenza pandemic in the United States. Pediatrics. 2011;128(6):e1450-8. PubMed PMID: 22065262; PubMed Central PMCID: PMCPMC3387899.

194. Rello J, Perez M, Roca O, Poulakou G, Souto J, Laborda C, et al. High-flow nasal therapy in adults with severe acute respiratory infection. A cohort study in patients with 2009 influenza A/H1N1v. Journal of Critical Care. 2012;27(5):434-9. PubMed PMID: 2012597869.

195. Rello J, Rodriguez A, Ibanez P, Socias L, Cebrian J, Marques A, et al. Intensive care adult patients with severe respiratory failure caused by Influenza A (H1N1)v in Spain. Critical Care (London, England). 2009;13(5):R148. PubMed PMID: 19747383; PubMed Central PMCID: PMCPMC2784367.

196. Rhedin S, Hamrin J, Naucler P, Bennet R, Rotzen-Ostlund M, Farnert A, et al. Respiratory viruses in hospitalized children with influenza-like illness during the h1n1 2009 pandemic in Sweden [corrected]. PLoS One. 2012;7(12):e51491. Epub 2012/12/29. doi: 10.1371/journal.pone.0051491. PubMed PMID: 23272110; PubMed Central PMCID: PMC3522717.

197. Rice TW, Thompson BT, Rubinson L, Uyeki T, Vaughn FL, John B, et al. Role of bacterial co-infection on illness severity and outcomes in ICU patients with 2009 pandemic influenza a (H1N1) infection. American Journal of Respiratory and Critical Care Medicine Conference: American Thoracic Society International Conference, ATS. 2011;183(1 MeetingAbstracts). PubMed PMID: 70845551.

198. Rios FG, Estenssoro E, Villarejo F, Valentini R, Aguilar L, Pezzola D, et al. Lung function and organ dysfunctions in 178 patients requiring mechanical ventilation during the 2009 influenza A (H1N1) pandemic. Critical Care (London, England). 2011;15(4):R201. PubMed PMID: 21849039; PubMed Central PMCID: PMCPMC3387643.

199. Riquelme R, Riquelme M, Rioseco ML, Inzunza C, Gomez Y, Contreras C, et al. Characteristics of hospitalised patients with 2009 H1N1 influenza in Chile. European Respiratory Journal. 2010;36(4):864-9. PubMed PMID: 2010566057.

200. Riquelme R, Torres A, Rioseco ML, Ewig S, Cilloniz C, Riquelme M, et al. Influenza pneumonia: a comparison between seasonal influenza virus and the H1N1 pandemic. European Respiratory Journal. 2011;38(1):106-11. PubMed PMID: 21109555.

201. Riscili BP, Anderson TB, Prescott HC, Exline MC, Sopirala MM, Phillips GS, et al. An assessment of H1N1 influenza-associated acute respiratory distress syndrome severity after adjustment for treatment characteristics. PLoS ONE [Electronic Resource]. 2011;6(3):e18166. PubMed PMID: 21464952; PubMed Central PMCID: PMCPMC3064596.

202. Roch A, Lepaul-Ercole R, Grisoli D, Bessereau J, Brissy O, Castanier M, et al. Extracorporeal membrane oxygenation for severe influenza A (H1N1) acute respiratory distress syndrome: A prospective observational comparative study. Intensive Care Med. 2010;36(11):1899-905. PubMed PMID: 2010644089.

203. Rodriguez A, Diaz E, Martin-Loeches I, Sandiumenge A, Canadell L, Diaz JJ, et al. Impact of early oseltamivir treatment on outcome in critically ill patients with 2009 pandemic influenza A. Journal of Antimicrobial Chemotherapy. 2011;66(5):1140-9. PubMed PMID: 21385717.

204. Roncon-Albuquerque R, Jr., Basilio C, Figueiredo P, Silva S, Mergulhao P, Alves C, et al. Portable miniaturized extracorporeal membrane oxygenation systems for H1N1-related severe acute respiratory distress syndrome: a case series. Journal of Critical Care. 2012;27(5):454-63. Epub 2012/03/06. doi: 10.1016/j.jcrc.2012.01.008. PubMed PMID: 22386225.

205. Sahoo JN, Poddar B, Azim A, Singh RK, Gurjar M, Baronia AK. Pandemic (H1N1) 2009 influenza: Experience from a critical care unit in India. Indian Journal of Critical Care Medicine. 2010;14(3):156-9. PubMed PMID: 2011016323.

206. Samra T, Pawar M, Yadav A. Comparative evaluation of acute respiratory distress syndrome in patients with and without H1N1 infection at a tertiary care referral center. Indian Journal of Anaesthesia. 2011;55(1):47-51. PubMed PMID: 2011246360.

207. Sasbon JS, Centeno MA, Garcia MD, Boada NB, Lattini BE, Motto EA, et al. Influenza A (pH1N1) infection in children admitted to a pediatric intensive care unit: differences with other respiratory viruses. Pediatric Critical Care Medicine. 2011;12(3):e136-40. PubMed PMID: 20431501.

208. Satterwhite L, Mehta A, Martin GS. Novel findings from the second wave of adult pH1N1 in the United States. Crit Care Med. 2010;38(10):2059-61. PubMed PMID: 20625279.

209. Schellongowski P, Ullrich R, Hieber C, Hetz H, Losert H, Hermann M, et al. A surge of flu-associated adult respiratory distress syndrome in an Austrian tertiary care hospital during the 2009/2010 Influenza A H1N1v pandemic. Wiener Klinische Wochenschrift. 2011;123(7-8):209-14. PubMed PMID: 21465083.

210. Scriven J, McEwen R, Mistry S, Green C, Osman H, Bailey M, et al. Swine flu: a Birmingham experience. Clinical Medicine. 2009;9(6):534-8. PubMed PMID: 20095293.

211. Sertogullarindan B, Ozbay B, Gunini H, Sunnetcioglu A, Arisoy A, Bilgin HM, et al. Clinical and prognostic features of patients with pandemic 2009 influenza a (H1N1) virus in the intensive care unit. African Health Sciences. 2011;11(2):163-70. PubMed PMID: 2011422912.

212. Shahpori R, Stelfox HT, Doig CJ, Boiteau PJE, Zygun DA. Sequential Organ Failure Assessment in H1N1 pandemic planning. Crit Care Med. 2011;39(4):827-32. PubMed PMID: 21263327.

213. Shlomai A, Nutman A, Kotlovsky T, Schechner V, Carmeli Y, Guzner-Gur H. Predictors of pandemic (H1N1) 2009 virus positivity and adverse outcomes among hospitalized patients with a compatible syndrome. Israel Medical Association Journal: Imaj. 2010;12(10):622-7. PubMed PMID: 21090520.

214. Siau C, Law J, Tee A, Poulose V, Raghuram J. Severe refractory hypoxaemia in H1N1 (2009) intensive care patients: initial experience in an Asian regional hospital. Singapore Medical Journal. 2010;51(6):490-5. PubMed PMID: 20658109.

215. Sood MM, Rigatto C, Zarychanski R, Komenda P, Sood AR, Bueti J, et al. Acute kidney injury in critically ill patients infected with 2009 pandemic influenza A(H1N1): report from a Canadian Province. American Journal of Kidney Diseases. 2010;55(5):848-55. PubMed PMID: 20303633.

216. Stein M, Tasher D, Glikman D, Shachor-Meyouhas Y, Barkai G, Yochai AB, et al. Hospitalization of children with influenza A(H1N1) virus in Israel during the 2009 outbreak in Israel: a multicenter survey. Arch Pediatr Adolesc Med. 2010;164(11):1015-22. Epub 2010/11/03. doi: 10.1001/archpediatrics.2010.195. PubMed PMID: 21041594.

217. Subramony H, Lai FYL, Ang LW, Cutter JL, Lim PL, James L. An epidemiological study of 1348 cases of pandemic H1N1 influenza admitted to Singapore Hospitals from July to September 2009. Annals of the Academy of Medicine, Singapore. 2010;39(4):283-8. PubMed PMID: 20473452.

218. Sun JJ, Li C, Wu DX, Li CH, Qin WH, Li YL, et al. Eighteen cases of 2009 influenza a H1N1 associated with respiratory failure in adults. [Chinese]. Chinese Critical Care Medicine. 2010;22(3):156-60. PubMed PMID: 2010267012.

219. Kumar S, Havens PL, Chusid MJ, Willoughby RE, Jr., Simpson P, Henrickson KJ. Clinical and epidemiologic characteristics of children hospitalized with 2009 pandemic H1N1 influenza A infection. Pediatric Infectious Disease Journal. 2010;29(7):591-4. PubMed PMID: 20589976.

220. Tabarsi P, Moradi A, Marjani M, Baghaei P, Hashemian S, Nadji S, et al. Factors associated with death or intensive care unit admission due to pandemic 2009 influenza A (H1N1) infection. Annals of Thoracic Medicine. 2011;6(2):91-5. PubMed PMID: 2011210379.

221. Teke T, Coskun R, Sungur M, Guven M, Bekci TT, Maden E, et al. 2009 H1N1 influenza and experience in three critical care units. International Journal of Medical Sciences. 2011;8(3):270-7. PubMed PMID: 21487571; PubMed Central PMCID: PMCPMC3074093.

222. Tokuhira N, Shime N, Inoue M, Kawasaki T, Sakurai Y, Kurosaka N, et al. Mechanically ventilated children with 2009 pandemic influenza A/H1N1: results from the National Pediatric Intensive Care Registry in Japan. Pediatric Critical Care Medicine. 2012;13(5):e294-8. PubMed PMID: 22805157.

223. Torres JP, O'Ryan M, Herve B, Espinoza R, Acuna G, Manalich J, et al. Impact of the novel influenza A (H1N1) during the 2009 autumn-winter season in a large hospital setting in Santiago, Chile. Clin Infect Dis. 2010;50(6):860-8. Epub 2010/02/20. doi: 10.1086/650750. PubMed PMID: 20166819.

224. Torres SF, Iolster T, Schnitzler EJ, Farias JA, Bordogna AC, Rufach D, et al. High mortality in patients with influenza A pH1N1 2009 admitted to a pediatric intensive care unit: a predictive model of mortality. Pediatric Critical Care Medicine. 2012;13(2):e78-83. PubMed PMID: 21552180.

225. Trimarchi H, Greloni G, Campolo-Girard V, Giannasi S, Pomeranz V, San-Roman E, et al. H1N1 infection and the kidney in critically ill patients. Journal of Nephrology. 2010;23(6):725-31. PubMed PMID: 20349409.

226. Turner DA, Rehder KJ, Peterson-Carmichael SL, Ozment CP, Al-Hegelan MS, Williford WL, et al. Extracorporeal membrane oxygenation for severe refractory respiratory failure secondary to 2009 H1N1 influenza A. Respiratory Care. 2011;56(7):941-6. PubMed PMID: 21352668.

227. Uchimura T, Mori M, Nariai A, Yokota S. Analysis of cases of severe respiratory failure in children with influenza (H1N1) 2009 infection in Japan. Journal of Infection & Chemotherapy. 2012;18(1):59-65. PubMed PMID: 21809061; PubMed Central PMCID: PMCPMC3278626.

228. Van Ierssel SH, Ieven M, Jorens PG. Severe influenza A(H1N1)2009 infection: A single centre experience and review of the literature. Acta Clinica Belgica. 2011;67(1):1-6. PubMed PMID: 2012347134.

229. van Zwol A, Witteveen R, Markhorst D, Geukers VGM. Clinical features of a Dutch cohort of critically ill children due to the 2009 new influenza A H1N1 pandemic. Clinical Pediatrics. 2011;50(1):69-72. PubMed PMID: 20837610.

230. Venkata C, Sampathkumar P, Afessa B. Hospitalized patients with 2009 H1N1 influenza infection: the Mayo Clinic experience. Mayo Clinic Proceedings. 2010;85(9):798-805. PubMed PMID: 20664021; PubMed Central PMCID: PMCPMC2931615.

231. Vidovic J, Kovacevic P, Stanetic M, Rajkovaca Z, Zlojutro B. Treatment of critically ill patients with influenza a H1N1 in university hospital Banja Luka. Acta Medica Saliniana. 2011;40(SUPPL. 1):S49-S51. PubMed PMID: 2011433906.

232. Pettila V, Webb SAR, Bailey M, Howe B, Seppelt IM, Bellomo R. Acute kidney injury in patients with influenzaA (H1N1) 2009. Intensive Care Med. 2011;37(5):763-7. PubMed PMID: 21394631.

233. Wiesen J, Komara JJ, Walker E, Wiedemann HP, Guzman JA. Relative cost and outcomes in the intensive care unit of acute lung injury (ALI) due to pandemic influenza compared with other etiologies: A single- center study. Annals of Intensive Care. 2012;2(1). PubMed PMID: 2012538252.

234. Wijaya L, Chua YY, Cui L, Chan K, Tan BH. Intravenous zanamivir in critically ill patients due to pandemic 2009 (H1N1) influenza A virus. Singapore Medical Journal. 2011;52(7):481-5. PubMed PMID: 21808957.

235. Wu JP, Wu Q, Du ZZ. Experiences in treatment of H1N1 pneumonitis with acute respiratory distress syndrome in Tianjin: A report of 9 cases. [Chinese]. Chinese Critical Care Medicine. 2010;22(3):166-8. PubMed PMID: 2010267015.

236. Yeung JH, Bailey M, Perkins GD, Smith FG. Presentation and management of critically ill patients with influenza A (H1N1): a UK perspective. Crit Care. 2009;13(6):426; author reply Epub 2009/12/04. doi: 10.1186/cc8151. PubMed PMID: 19954506; PubMed Central PMCID: PMC2811938.

237. Yu H, Feng Z, Uyeki TM, Liao Q, Zhou L, Feng L, et al. Risk factors for severe illness with 2009 pandemic influenza A (H1N1) virus infection in China. Clinical Infectious Diseases. 2011;52(4):457-65. PubMed PMID: 21220768; PubMed Central PMCID: PMCPMC3060897.

238. Yung M, Slater A, Festa M, Williams G, Erickson S, Pettila V, et al. Pandemic H1N1 in children requiring intensive care in Australia and New Zealand during winter 2009. Pediatrics. 2011;127(1):e156-e63. PubMed PMID: 2011008316.

239. Zhang PJ, Li XL, Cao B, Yang SG, Liang LR, Gu L, et al. Clinical features and risk factors for severe and critical pregnant women with 2009 pandemic H1N1 influenza infection in China. BMC Infectious Diseases. 2012;12:29. PubMed PMID: 22292815.

240. Zhang Q, Ji W, Guo Z, Bai Z, MacDonald NE. Risk factors and outcomes for pandemic H1N1 influenza compared with seasonal influenza in hospitalized children in China. Canadian Journal of Infectious Diseases and Medical Microbiology. 2012;23(4):199-203. PubMed PMID: 2013059009.

241. Zhao C, Gan Y, Sun J. Radiographic study of severe Influenza-A (H1N1) disease in children. European Journal of Radiology. 2011;79(3):447-51. PubMed PMID: 20965678.

242. Zimmerman O, Rogowski O, Aviram G, Mizrahi M, Zeltser D, Justo D, et al. C-reactive protein serum levels as an early predictor of outcome in patients with pandemic H1N1 influenza A virus infection. BMC Infectious Diseases. 2010;10:288. Epub 2010/10/06. doi: 10.1186/1471-2334-10-288. PubMed PMID: 20920320; PubMed Central PMCID: PMC2959060.

243. Carrillo-Esper R, Sosa-Garcia JO, Arch-Tirado E. [Experience in the management of the severe form of human influenza A H1N1 pneumonia in an intensive care unit]. Cirugia y Cirujanos. 2011;79(5):409-16. PubMed PMID: 22385760.

244. Topfer L, Menk M, Weber-Carstens S, Spies C, Wernecke KD, Uhrig A, et al. Influenza A (H1N1) vs non-H1N1 ARDS: analysis of clinical course. J Crit Care. 2014;29(3):340-6. doi: 10.1016/j.jcrc.2013.12.013. PubMed PMID: 24508203.

245. Rohani P, Jude CM, Chan K, Barot N, Kamangar N. Chest Radiological Findings of Patients With Severe H1N1 Pneumonia Requiring Intensive Care. J Intensive Care Med. 2016;31(1):51-60. doi: 10.1177/0885066614538753. PubMed PMID: 24923491.

246. Payet C, Lutringer-Magnin D, Cassier P, Lina B, Argaud L, Allaouchiche B, et al. [Description of patients with confirmed influenza A(H1N1)pdm09 admitted to an intensive care unit and identification of severity risk factors]. Med Mal Infect. 2013;43(2):81-4. doi: 10.1016/j.medmal.2013.01.016. PubMed PMID: 23453670.

247. Rao S, Torok MR, Bagdure D, Cunningham MA, Williams JT, Curtis DJ, et al. A comparison of H1N1 influenza among pediatric inpatients in the pandemic and post pandemic era. J Clin Virol. 2015;71:44-50. doi: 10.1016/j.jcv.2015.07.308. PubMed PMID: 26370314.

248. Nermin KG, Remzi I, Zeynep A, Ilker C, Meltem OT, Guven O, et al. A retrospective evaluation of critically ill patients infected with H1N1 influenza A virus in Bursa, Turkey, during the 2009-2010 pandemic. Afr Health Sci. 2015;15(2):352-9. doi: 10.4314/ahs.v15i2.7. PubMed PMID: 26124779; PubMed Central PMCID: PMCPMC4480494.

249. Funk DJ, Kumar A. Inhaled nitric oxide in patients with the acute respiratory distress syndrome secondary to the 2009 influenza A (H1N1) infection in Canada. Can J Anaesth. 2013;60(2):212-3. doi: 10.1007/s12630-012-9848-8. PubMed PMID: 23224680.

250. Cruz-Lagunas A, Jimenez-Alvarez L, Ramirez G, Mendoza-Milla C, Garcia-Sancho MC, Avila-Moreno F, et al. Obesity and pro-inflammatory mediators are associated with acute kidney injury in patients with A/H1N1 influenza and acute respiratory distress syndrome. Exp Mol Pathol. 2014;97(3):453-7. doi: 10.1016/j.yexmp.2014.10.006. PubMed PMID: 25305354.

251. Thompson SG, Higgins JP. How should meta-regression analyses be undertaken and interpreted? Stat Med. 2002;21(11):1559-73. Epub 2002/07/12. doi: 10.1002/sim.1187. PubMed PMID: 12111920.

**Table S8: List of excluded studies**

| **No.** | **Study Identifier** | **Country of Study** | **Reason for Exclusion** |
| --- | --- | --- | --- |
| 1. | Azziz- Baumgartner, PLoS One, 2012 | Argentina | Discusses the burden of disease and resource utilization associated with H1N1 and does not focus upon patient level variables |
| 2. | Palacios, PlosOne, 2009 | Argentina | Severity of illness did not meet our inclusion criteria |
| 3. | Trimarchi, NDT plus 2009 | Argentina | Detailed findings of the same population described in another manuscript |
| 4. | Kusznierz, Influenz and other respir viruses, 2013 | Argentina | Mortality in critically ill patients not described |
| 5. | Forrest, Intensive Care Medicine, 2011 | Aus/NZ | Discusses only transportation of patients requiring ECMO |
| 6. | Fitzgerald, Crit Care and Resuscitation, 2012 | Aus/NZ | Letter to the editor; discusses the difficulties with continuous veno-venous hemodialysis in patients undergoing HFOV |
| 7. | Hayashi, Internal Medicine Journal, 2011 | Aus/NZ | No clear distinction of critically ill patients from other patients |
| 8. | Ng, American Journal of Transplantation, 2011 | Aus/NZ | Fewer than 5 critically ill patients |
| 9. | Bellomo, Contributions to Nephrology, 2010 | Australia | Outcome variables of interest not described |
| 10. | Mulrennan, PLoS One, 2010 | Aus/NZ | Outcome variables of interest not described |
| 11. | Hodgson, Crit Care, 2012 | Australia | Described only long term quality of life in ECMO patients, not outcomes of interest |
| 12. | Higgins, Anaesth Intensive Care, 2011 | Aus/ NZ | Discusses the economic impact of H1N1 Pandemic |
| 13. | Hewagama, Clin Infect Disease, 2010 | Aus/ NZ | No data describing critically ill patients provided |
| 14. | Burns, Prehospital Emergency Care, 2011 | Australia | Discusses logistics of ECMO retrieval |
| 15. | Pirakalathanan, Journal of Medical Imaging and Radiation Oncology, 2013 | Australia | Only discusses the radiographic findings in H1N1 patients |
| 16. | Lum, Medical Journal of Australia, 2009 | Australia | Modeling study to examine the demands associated with critical care services during the H1N1 pandemic |
| 17. | Khandaker, Neurology 2012 | Australia | Neurologic findings associated with H1N1 in pediatric patients; no clearly defined parameters for critically ill children |
| 18. | Li, Chinese Medical Journal, 2012 | China | Describes only histopathological findings |
| 19. | Capelozzi, Clinics, 2010 | Brazil | Describes only morphological features associated with ARDS in H1N1 |
| 20. | Seixas, Histopathology 2010 | Brazil | Describes histopathology in fatal cases |
| 21. | Lorenzoni, Arquivos de Neuro-Psiquiatria, 2012 | Brazil | Describes muscle biopsy results in only fatal cases |
| 22. | Lenzi, Revista Da Sociedade Brasileira de Medicina Tropical | Brazil | No outcomes associated with critical illness reported separately |
| 23. | Morris, BMJ Open, 2012 | Canada | No mortality in critically ill patients provided |
| 24. | Muller, PLoS One, 2010 | Canada | Has non-H1N1 data |
| 25. | Campbell, CMAJ, 2010 | Canada | Death and ICU admission not described separately |
| 26. | Helferty, CMAJ, 2010 | Canada | No ICU outcomes described |
| 27. | Zahariadis, Infect Dis Med Microbiol, 2010 | Canada | Only two patients described, otherwise a review of microbiology and genetics of H1N1 |
| 28. | Zhang, Chinese Medical Journal, 2012 | China | No clinical outcomes described |
| 29. | Fang, PLos One, 2012 | China | No clinical outcomes described |
| 30. | Xu, PLos One, 2013 | China | Post-pandemic cohort described |
| 31. | Yang, Journal of Infection, 2010 | China | Separate outcomes of critically ill patients not described |
| 32. | Yan, Chinese Journal of Internal Medicine, 2009 | China | Critically ill patients not described |
| 33. | Chen, Chinese Journal of Radiology | China | No clinically relevant outcomes discussed |
| 34. | Wu, national Medical Journal of China | China | Only discusses the features of fatal cases |
| 35. | Leick-Courtois, Archives de Pediatrie, 2011 | France | Fewer than 5 critically ill patients |
| 36. | Luyt, Chest, 2012 | France | Discusses Long-term outcomes in ARDS patients |
| 37. | Annane, Intensive Care Medicine, 2012 | France | No outcomes of interest are described |
| 38. | Fuhrman, Eurosurveillance, 2010 | France | Outcomes in critically ill patients not described separately |
| 39. | Wiramus, Annales Francaises d’Anesthesie et de Reanimation, 2010 | France | Reviews epidemiological data from different studies throughout the world, no new data presented |
| 40. | Gonzalo-Morales, Rev Chil Pediatr 2011 | Chile | Characteristics and outcomes associated with critical illness not clearly mentioned |
| 41. | Ugarte, Crit Care Med, 2010 | Chile | No patient specific data of interest provided |
| 42. | Gudmundsson, Laeknabladid, 2010 | Iceland | Editorial |
| 43. | Prasad, The Journal of the association of Physicians of India | India | Only describes autopsy findings |
| 44. | Bal, Histopathology, 2012 | India | Only describes autopsy findings |
| 45. | Sharma, Journal of Infect Dev Ctries 2010 | India | No information on critically ill patients |
| 46. | Shelke, Pathology International, 2012 | India | Only pathological findings described |
| 47. | Mishra, PLoSOne, 2010 | India | Does not describe any critically ill patients separately |
| 48. | Kute, Indian Journal of Critical Care, 2011 | India | Letter to the editor |
| 49. | Chudasama, Lung India, 2011 | India | No outcomes in critically ill patients reported |
| 50. | Chudasama, J Infect Dev Countries, 2010 | India | No outcomes associated with critical illness reported |
| 51. | Samra, Anaesth, Pain and Intensive Care, 2010 | India | Fewer than 5 patients |
| 52. | Samra, Indian J Community Med, 2011 | India | Letter to the editor, not describing variables associated with critical illness |
| 53. | Kinikar, Indian J Pediatr, 2011 | India | No variables associated with critical illness described |
| 54. | Kinikar, Indian J Pediatr, 2012 | India | No variables associated with critical illness described |
| 55. | Jahromi, International Journal of Obstetric Anesthesia, 2010 | Iran | Fewer than 5 patients |
| 56. | Gouya, Iranian Red Crescent Medical Journal | Iran | No variables associated with critical illness were discussed |
| 57. | Saleh, Iranian Journal of Clinical Infectious Diseases | Iran | No outcomes associated with critical illness reported |
| 58. | Baldanti, Clin Microbiol Infect 2011 | Italy | Doesn’t describe specific information in critically ill patients |
| 59. | Bellissima, Le Infezioni in Medicina, 2011 | Italy | Fewer than 5 patients |
| 60. | NIcolini, Rev Port Pneumol, 2012 | Italy | No clinical outcomes of interest described in the text |
| 61. | Valente, Radiol Med, 2012 | Italy | No clinical outcomes of Interest described in the text |
| 62. | Okumura, Brain and Development, 2012 | Japan | Critically ill population not defined |
| 63. | Nukiwa, Clinical Infect Dis, 2010 | Japan | Only fatal cases described |
| 64. | Lopez, Med Intensiva, 2009 | Spain | Case Report |
| 65. | Chippiraz, Rev Esp Quimioter, 2011 | Spain | Patients described in the study have very low APACHE score, so they were excluded |
| 66. | Pinilla, Emerg Radiol 2011 | Spain | No clinical outcomes associated with critical illness reported |
| 67. | Martin-Loeches, Respirology, 2011 | Spain | Describes only fatal cases in Spain |
| 68. | Peralta, Eurosurveillance 2010 | Spain | Describes death and ICU admission as a combined outcome without a mechanism to disaggregate |
| 69. | Gutierrez-Cuadra, Revista Espanola de Quimioterapia | Spain | No data associated with critical illness provided |
| 70. | Rodriguez, Medicina Intensiva, 2011 | Spain | Describes the outcomes associated with ICU admissions in the post pandemic period |
| 71. | Cardenosa, Human Vaccines, 2011 | Spain | Variables associated with critical illness not described separately from hospitalized patients |
| 72. | Gonzalez, Enfermedades Infecciosas y Microbiologia Clinica, 2011 | Spain | No specific variables associated with critical illness described separately |
| 73. | Viasus, Clinical Microbiology and Infection, 2011 | Spain | ICU admission and mortality were used as a composite measure for severe disease |
| 74. | Rodriguez, Archivos de Bronchoneumologia, 2010 | Spain | Review article |
| 75. | Bibro, Critical Care Nurse, 2011 | USA | Case report |
| 76. | Nickel, Public Health Reports 2011 | USA | Describes death and ICU admission together with no mechanism to disaggregate |
| 77. | Fowlkes, Clinical Infectious Disease, 2011 | USA | Only describes the epidemiology of fatal cases in USA |
| 78. | Strouse, Blood, 2010 | USA | No information on critically ill patients |
| 79. | Farooq, J Child Neurol, 2012 | USA | Outcomes in critically ill patients are not separately reported |
| 80. | McKenna, BMC Infectious Diseases, 2013 | USA | Describes death and ICU admission together |
| 81. | Mendez-Figueroa, Am J Obstet Gynecol, 2011 | USA | Only 3 patients admitted to the neonatal ICU |
| 82. | Jain, Clinical Infectious Diseases 2012 | USA | Same population was reported in article by Bramley et al |
| 83. | Skarbinski, Clinical Infectious Diseases, 2011 | USA | Same population was reported in article by Bramley et al |
| 84. | Regan, Influenza 2011 | USA | Only describes the epidemiology of fatal cases in USA |
| 85. | Cox, Clinical Infectious Diseases, 2011 | USA | Only has information on pediatric fatalities during the H1N1 pandemic |
| 86. | Lee, Clinical Infectious Diseases, 2011 | USA | Only has information on fatal cases in New York |
| 87. | Louie, PLoS ONE, 2011 | USA | Only describes fatal cases in California |
| 88. | Nguyen, Crit Care Medicine, 2012 | USA | No information on mortality in the entire cohort of patients |
| 89. | Michaels, American Journal of Surgery, 2013 | USA | Only characteristics of ECMO discussed in this article |
| 90. | Miller, Journal of Intensive Care Medicine, 2011 | USA | No outcomes of interest reported |
| 91. | Sundar, Journal of Intensive care Medicine, 2011 | USA | Variables all divided into short term and long term mechanical ventilation |
| 92. | Newsome, Birth Defects Research | USA | Only outcomes of Infants of critically ill pregnant females |
| 93. |  |  |  |
| 94. | Li, Journal of Clinical Virology, 2009 | USA | Uses all patients infected with different strains of influenza |
| 95. | Katouzian, Journal of Investigative Medicine, 2010 | USA | No outcomes of interest discussed |
| 96. | Pannaraj, Journal of Perinatology, 2011 | USA | No outcomes of interest were described |
| 97. | Jamieson, Lancet, 2009 | USA | Critically ill patients not described separately |
| 98. | Valdes, Rev Cubana Med Trop, 2011 | Cuba | Critically ill patients not described separately |
| 99. | Molbak, Vaccine, 2011 | Denmark | ICU specific outcomes not described |
| 100. | Ahmed, Influenza and other respiratory viruses, 2011 | Egypt | Outcomes associated with critical illness not described |
| 101. | Bauernfiend, Infection, 2013 | Germany | Influenza A H1N1patients not clearly defined as compared to infection due to other viruses |
| 102. | Lehners, Emerging Infectious Diseases, 2013 | Germany | ICU admission and mortality were reported together as a marker for severe disease |
| 103. | Stein, Klin Pediatr 2011 | Germany | Reports only on premature neonates |
| 104. | Burkle, Anaesthesist 2010 | Germany | Outcomes associated with critical illness not described clearly |
| 105. | Alb, Dtsch Med Wochenschr, 2010 | Germany | Outcomes associated with critical illness not described clearly |
| 106. | Zarogoulidis, International Journal of Internal Medicine, 2013 | Greece | Outcomes associated with critical illness not reported separately |
| 107. | Lee, The Journal of Infectious Diseases, 2011 | Hong Kong | Outcomes associated with critical illness not reported separately |
| 108. | Lee, Thorax, 2013 | Hong Kong | Specific characteristics and outcomes associated with critical illness not reported separately |
| 109. | Sigurdsson, Laekna, 2010 | Iceland | Outcomes associated with critical illness not reported clearly |
| 110. | Bayya- Ael, Crit Care and Resuscitation, 2010 | Israel | No outcomes reported |
| 111 | Shaham, IMAJ, 2011 | Israel | No outcomes of interest reported |
| 112. | Saidel-Odes, International Journal of Infectious Diseases, 2011 | Israel | Critically ill population not clearly delineated |
| 113. | Takeda, Journal of Anesthesia, 2012 | Japan | Majority of the patients included in the study were in the post pandemic phase |
| 114. | Fuchigami, Pediat Emergency Med 2012 | Japan | Critically ill patients not reported separately |
| 115. | Okada, J Infect Chemother, 2011 | Japan | Patients did not meet our definition for critical illness |
| 116. | Fujita, Influenza and other respiratory viruses, 2011 | Japan | Letter to the editor |
| 117. | Wada, Influenza and other respiratory viruses, 2010 | Japan | ICU admission and mortality were described as a composite variable with no mechanism to disaggregate |
| 118. | Choi, Tuberc Respir Dis 2010 | South Korea | Critically ill specific outcomes not described |
| 119. | Na, Scandinavian Journal of Infectious Diseases, 2011 | South Korea | Critically ill specific population not defined |
| 120. | Goong, Infection and Chemotherapy | South Korea | Critically ill specific population not described |
| 121. | Balraj, Malaysian Journal of pathology, 2011 | Malaysia | Patient characteristics and outcomes not described |
| 122. | Chowell, NEJM, 2009 | Mexico | No characteristics or outcomes associated with critical illness described |
| 123. | Echevarria-Zuno, Lancet, 2009 | Mexico | Critically ill patients not described separately |
| 124. | Vazquez- Perez, Virology Journal, 2011 | Mexico | Critically ill patients not described separately |
| 125. | Chowell, PLos One, 2012 | Mexico | Critically ill patients not described separately |
| 126. | Silva-Pereya, NEJM, 2009 | Mexico | Only pathological findings described |
| 127. | Rahamat- Langendoen, Journal of Clinical Virology 2012 | Netherlands | Critically ill patients not described separately |
| 128. | Pajankar, Oman Medical Journal, 2012 | Oman | Patients were not sick enough to be considered critically ill |
| 129. | Rorat, Postepy HIg Med Dosw, 2013 | Poland | Only describes fatal cases |
| 130. | Cholewinska, Przeglad Epidemiologiczny, 2010 | Poland | Critically ill patient outcomes not described separately |
| 131. | Agha, Mediterranean Journal of Hematology and Infectious Diseases, 2012 | Saudi Arabia | Critically ill patients not described separately |
| 132. | Liu, Chin Crit Care Med, 2010 | China | Only risk factors for critical illness discussed, no outcomes associated with critical illness were described |
| 133. | Siau, Singapore Medical Journal, 2009 | Singapore | Critically ill patients not described |
| 134. | Wiegand, Wein Klin Wochenschr, 2011 | Switzerland | Fewer than 5 patients |
| 135. | Bertisch, Swiss Med Wkly, 2010 | Switzerland | Critically ill patients not described |
| 136. | Dede, BJOG, 2011 | Turkey | Only describes maternal deaths associated with H1N1 |
| 137. | Ozkan, Pediatric Neurology, 2011 | Turkey | Critically ill specific cases are not described |
| 138. | Gurgun, Tuberkuloz ve Toraks Dergisi, 2010 | Turkey | Patients were not sick enough to qualify to be considered critically ill |
| 139. | Tutuncu, Saudi Med J, 2010 | Turkey | Discusses risk factors associated with mortality |
| 140. | Lucas, Health technology Assessment, 2010 | UK | Only discusses fatal cases |
| 141. | Mytton, Eurosurveillance, 2012 | UK | No specific outcomes associated with critical illness reported |
| 142. | Campbell, Epidemiol. Infect 2011 | UK | No outcomes associated with critical illness reported |
| 143. | Mytton, Epidemiol. Infect, 2012 | UK | No specific characteristics or outcomes associated with critical illness described |
| 144. | Brett, PLos One 2011 | UK | ICU admission and death considered as a combined outcome |
| 145. | Bewick, Thorax, 2011 | UK | Outcomes associated with critical illness not reported |
| 146. | Myles, PLoS One, 2012 | UK | Outcomes associated with critical illness not reported |
| 147. | Myles, Thorax, 2012 | UK | ICU admission and death considered as a composite outcome |
| 148. | Khan, Anaesthesia, 2009 | UK | Only assesses validity of SOFA score as a triage tool |
| 149. | Fox, PLoS One 2012 | Vietnam | No separate data on critically ill patients |
| 150. | Wang, Chin Crit Care Med, 2010 | China | Only 4 patients described |
| 151. | Kato, Nippon Rinsho- Japanese Journal of Clinical Medicine | Japan | Review Article |
| 152. | Guler Ozturk | Turkey | Describes only 4 patients |
| 153. | Dalziel, BMJ 2013 |  | Critical illness and mortality were considered as a composite outcome |
| 154. | Jamieson, Lancet, 2009 | USA | Outcomes associated with critical illness not reported separately |
| 155. | Evdokimov, Anesteziologiia i Reanimatologiia, 2010 | Russia | Full text not available |
| 156. | Dabnach, Emerging Infectious Diseases, 2011 | Chile | Specific characteristics associated with critical illness not discussed |
| 157. | Olga, Anesteziologie e Intenzivni Medicina, 2010 | Czech | Full text article not available |
| 158. | Oersted, Clin Microbiology and Infection, 2012 | Denmark | No outcomes associated with critical illness discussed |
| 159. | Snacken, Influenza and Other Respiratory Viruses | Multiple Countries | Outcomes associated with critical illness not described clearly |
| 160. | Nakashidze, Georgian Medical News 2012 | Georgia | Outcomes associated with critical illness not reported separately |
| 161. | Chowell, NEJM, 2009 | Mexico | Critically ill population not described clearly |
| 162. | Firstenberg, Emerging Infectious Diseases, 2009 | USA | Case Report |
| 163. | Gomez, Eurosurveillance, 2009 | Peru | Critically ill population not described |
| 164. | Grijalva- Otero, Archives of Medical Research, 2009 | Mexico | Describes only fatal cases |
| 165. | Moreno, Intensive Care Medicine, 2009 | NA | Review |
| 166. | Oliveira, Eurosurveillance, 2009 | Brazil | Characteristics associated with critical illness not described separately |
| 167. | Fowler, Crit Care Med, 2010 | NA | Review article |
| 168. | Patel, Anaesthesia, 2009 | UK | Fewer than 5 patients |
| 169. | Peters, Deutsches Arzteblatt, 2009 | Germany | Editorial |
| 170. | Smetanin, Canadian Journal of Infectious Diseases and Medical Microbiology, 2009 | Canada | Patient level variables and outcomes not described |
| 171. | Presanis, PLoS Medicine, 2009 | USA | Bayesian Model evaluating severity associated with H1N1 |
| 172. | Taran, Revista de la Facultad de Ciencias Medicas de Corboda, 2009 | Argentina | Critically ill patients not described separately |
| 173. | Webb, Critical care and Resuscitation | Australia | Editorial |
| 174. | Akritidis, American Journal of Cardiology, 2010 | Greece | Critically ill patients not described separately |
| 175. | Allard, Diabetes Care, 2010 | Canada | Critically ill patients not described separately |
| 176. | Bellani, Intensive Care Unit, 2010 | Italy | Case Report |
| 177. | Berryman, Nursing in Critical Care, 2010 | UK | Case Report |
| 178. | Chitnis, WMJ, 2010 | USA | Critically ill patients not described separately |
| 179. | Castilla, Euro Surveillance, 2010 | Spain | Critically ill patients not described |
| 180. | Chiumello, IntensiveCare Medicine, 2010 | Italy | Outcomes associated with critical illness not discussed |
| 181. | He, Journal of Central South University, 2010 | China | Outcomes associated with critical illness not described |
| 182. | Derdak, Crit Care Medicine, 2010 | USA | No patient data given |
| 183. | Jaber, Annales Francaises d’ Anesthesie et de Reanimation, 2010 | France | Review Article |
| 184. | Jardim, Early Human Development, 2010 | Portugal | Patients did not meet our critically ill definition |
| 185. | Morgan, PLoS ONE, 2010 | USA | Outcomes associated with critical illness not described |
| 186. | Schoub, Expert Review of Respiratory Medicine, 2010 | South Africa | Review |
| 187. |  |  |  |
| 188. | Staudinger, Wiener Klinische Wochenschrift, 2010 | Austria | Review article |
| 189. | Weiss, Pneumologie, 2010 | Germany | Review Article |
| 190. | Bahloul, Trends in Anaesthesia and Critical Care, 2010 | Tunisia | Review Article |
| 191. | Charu, CID, 2011 | Mexico | Only fatal cases discussed |
| 192. | Falagas, Epidemiology and Infection, 2011 | Argentina | Review Article |
| 193. | Fezeu, Obesity reviews, 2011 | France | Systematic Review |
| 194. | Mosby, American Journal of Obstetrics and Gynecology, 2011 | USA | Systematic Review |
| 195. | Presanis, BMJ, 2011 | UK | Mathematical model of severity |
| 196. | Van Kerkhove, Influenza and other Respiratory viruses, 2011 | Multiple Countries | No outcomes associated with critical illness described |
| 197. | Van Kerkhove, PLoS ONE, 2011 | Multiple Countries | No outcomes associated with critical illness reported |
| 198. | Wong, Perfusion, 2011 | NA | Review |
| 199. | Barai, Australasian Medical Journal, 2012 | India | Outcomes associated with critical illness not described |
| 200. | Berdai, Pan African Medical Journal, 2012 | Morocco | Outcomes associated with critical illness not described |
| 201. | Dawood, The Lancet Infectious Diseases, 2012 | Multiple Countries | Outcomes associated with critical illness not described |
| 202. | Dubrov, Intensive Care Medicine, 2011 | Ukraine | Abstract only |
| 203. | Fernandez, Medicina Clinica, 2012 | NA | Post pandemic report |
| 204. | Homaira, Bulletin of WHO, 2012 | Bangladesh | Variables associated with critical illness not described |
| 205. | Roll, Infection, 2012 | Germany | Critically ill patients not described |
| 206. | Rolland- Harris, Epidemiology and Infection, 2012 | Canada | Critically ill patients not described |
| 207. | Schuck-Paim, PLoS ONE, 2012 | Brazil | Critically ill patients not described |
| 208. | Kuchar, Respiratory Physiology and Neurobiology | Poland | Critically ill patients not described |
| 209. | Marzano, Journal of Medical Virology, 2013 | Italy | Critically ill patients not described |
| 210. | Golokhvastova, Klinicheskaia Meditsina , 2012 | Russia | Full text not available |
| 211. | Iatyshina, Terapevticheskii Arkhiv, 2010 | Russia | Full text not available |
| 212. | Klimova, Terapevticheskii Arkhiv, 2010 | Russia | Full Text Not available |
| 213. | Kolobukhina, Terapevticheskii Arkhiv, 2011 | Russia | Full text not available |
| 214. | Luzina,Klinicheskaia Meditsina, 2011 | Russia | Full text not available |
| 215. | Morton, British Journal of Anesthesia, 2015 | UK, Canada | Post-pandemic Patients were not described separately |
| 216. | Meltzer, Clinical Infectious Diseases, 2015 | USA | Patient specific outcomes not described |
| 217. | Rovina, Respiratory Care, 2014 | Greece | Outcomes of Critically ill patients not described |
| 218. | Reed, Clinical Infectious Diseases, 2014 | USA | Critically ill patients not described |
| 219. | Fujikura, Japanese Journal of Infectious Diseases, 2014 | Japan | Results based on a survey of critical care physicians |
| 220. | Ali, Scandinavian Journal of Infectious Diseases, 2013 | Pakistan | Critically ill patients not described |
| 221. | Scotta, Jornal de Pediatria, 2013 | Brazil | Outcomes of Critically ill patients not described |
| 222. | Chang, Journal of Obstetrics and Gynaecology, 2013 | China | Only neonatal outcomes described |
| 223. | Bauernfeind, Infection, 2013 | Germany | Outcomes associated with critical illness not described separately |
| 224. | Ismail, International Medical Journal, 2015 | Malaysia | Critically ill patients not described |
| 225. | Rojas-Suarez, Journal of Perinatal Medicine, 2014 | Colombia | Critically ill patients not described separately |
| 226. | Welch, Intensive Care Medicine, 2015 | USA | Post-pandemic influenza patients included |
